# Hyphal growth determines spatial organization and coexistence in a pathogenic polymicrobial community in a spatially structured environment

**DOI:** 10.1093/ismejo/wraf279

**Published:** 2025-12-18

**Authors:** Leonardo Mancini, Laila Saliekh, Rory Claydon, Jurij Kotar, Eva Bernadett Benyei, Carol A Munro, Tyler N Shendruk, Aidan Brown, Martin Welch, Pietro Cicuta

**Affiliations:** Department of Physics, Cavendish Laboratory, University of Cambridge, J.J. Thomson Avenue, Cambridge, Cambridgeshire CB3 0HE, United Kingdom; Department of Biochemistry, University of Cambridge, Tennis Court Road, Cambridge, Cambridgeshire CB2 1QW, United Kingdom; School of Physics and Astronomy, The University of Edinburgh, Peter Guthrie Tait Road, Edinburgh, Midlothian EH9 3FD, United Kingdom; School of Physics and Astronomy, The University of Edinburgh, Peter Guthrie Tait Road, Edinburgh, Midlothian EH9 3FD, United Kingdom; Department of Physics, Cavendish Laboratory, University of Cambridge, J.J. Thomson Avenue, Cambridge, Cambridgeshire CB3 0HE, United Kingdom; Department of Biochemistry, University of Cambridge, Tennis Court Road, Cambridge, Cambridgeshire CB2 1QW, United Kingdom; Institute of Medical Sciences, University of Aberdeen, Foresterhill, Aberdeen, Aberdeenshire AB25 2ZD, United Kingdom; School of Physics and Astronomy, The University of Edinburgh, Peter Guthrie Tait Road, Edinburgh, Midlothian EH9 3FD, United Kingdom; School of Physics and Astronomy, The University of Edinburgh, Peter Guthrie Tait Road, Edinburgh, Midlothian EH9 3FD, United Kingdom; Department of Biochemistry, University of Cambridge, Tennis Court Road, Cambridge, Cambridgeshire CB2 1QW, United Kingdom; Department of Physics, Cavendish Laboratory, University of Cambridge, J.J. Thomson Avenue, Cambridge, Cambridgeshire CB3 0HE, United Kingdom

**Keywords:** microbial ecology, infection, soft matter, Pseudomonas aeruginosa, Staphylococcus aureus, Candida albicans, microenvironmental structure, mechanical interactions, microfluidics, pathogen coexistence

## Abstract

The bodies of macroorganisms host microbes living in multispecies communities. Sequencing approaches have revealed that different organs host different microbiota and tend to be infected by different pathogens, drawing correlations between environmental parameters at the organ level and microbial composition. However, less is known about the microscale dimension of microbial ecology, particularly during infection. In this study, we focus on the role of microscale spatial structure, studying its influence on the ecology of a polymicrobial infection of *Pseudomonas aeruginosa*, *Staphylococcus aureus*, and *Candida albicans*. Although these pathogens are commonly found together in the lungs of chronically ill patients, it is unclear whether they coexist or compete and segregate in different niches. We find that, whereas *P. aeruginosa* quickly outcompetes *C. albicans* and *S. aureus* on large surfaces, robust spatial organization and coexistence emerges in spatially structured microenvironments. In confined spaces, slowly growing *C. albicans* is able to leverage rapid radial hyphal growth to conquer boundaries, where it establishes itself displacing the other pathogens. Similar outcomes are observed when the *P. aeruginosa* strain carries *mexT*-inactivating mutations, which are often found in clinical isolates. The observed spatial organization enables coexistence and potentially determines infection severity and outcomes. Our findings reveal a previously unrecognized role of mechanical forces in shaping infection dynamics, suggesting that microenvironmental structure might be a critical determinant of pathogen coexistence, virulence, and treatment outcomes. Because adaptations, such as changes in morphology, are widespread among microbes, these results are generalizable to other ecologies and environments.

## Introduction

“Omic” studies have revealed that microbes seldom live in isolation, both in the healthy [1–3] and diseased body [4–6]. Instead, many different species come together to consume substrates giving rise to polymicrobial communities and rich ecologies [7–9]. Interactions with different structural characteristics of their environments produce spatial patterning that in “macroorganisms” we commonly call biogeography [10]. This patterning on both the macro and microscale eventually determines the phenotypic outcome of the polymicrobial community, either in terms of virulence [11–17], survival to treatment [17–21], or substrate transformation [21–24].

Although we have an understanding of macroscale factors due to “omic” approaches, microscale ones such as biotic factors and ecological interactions require highly spatially and time resolved assays [25], which until recently have been hard to perform with the same techniques [26]. These have been studied mainly through mathematical modeling [27, 28] or in synthetic communities grown in the lab [29–33]. In addition to revealing a wealth of new ecology, these “synthetic” communities have the merit of enabling live direct observations.

Microscopy complements omic approaches, offering high spatial and time resolution and an access to mechanistic understanding of microscale phenomena. Despite progress in biomedical imaging, the observation of these microbial consortia *in vivo* in humans remains a challenging task. Some indications on the shapes and forms of these communities are provided by *ex vivo* observations. These images have been fundamental to understanding that microbes can indeed have complex spatial organizations at the microscale—an “urban biogeography”—that can have significant consequences on their pathogenicity and biophysical and metabolic properties [22, 34]. Understanding how specific “urban biogeographies” come about and mapping different organizations to their emergent properties will open up new intervention strategies aimed at preventing and treating pathogenic scenarios, including infection. To achieve this goal, approaches that encompass the host, polymicrobial communities, live microscopy, and controlled environments are needed. Currently, two model scenarios satisfy these requirements in different measures and their choice depends on the research question: animal models and infection-on-chip. Animal models offer the most realistic conditions as they respond to infection with a complete immune system and therefore are well suited to study the complex interaction between microbes and host response [35–37]. However, imaging infection in these systems is challenging [38]. Even in transparent hosts, such as zebrafish larvae, the spatial resolution for live microscopy is limited to the organ level [39–45]. Organoids and infection-on-chip systems that encompass host tissues strike a compromise, improving imaging to the detriment of the immune response, which tends to be only partial [46–51]. In both cases, complexity remains high and isolating the mechanistic contribution of specific parameters can be challenging.

In this work, we focus on the role of spatial structure in the lung on shaping the microscale ecology of a respiratory pathogenic polymicrobial community. A large body of work [52–54] has demonstrated that microbial phenotype and evolution are conditioned by the mechanical properties of the substrate, both passively [55] and through active sensing [56, 57]. Contact with surfaces is commonly associated with the formation of mono- and polymicrobial biofilms [58, 59], which are studied almost exclusively on surfaces that do not impose limitations in the surface plane [60]. In these settings, spatial organization within biofilms has been studied as an emergent property of microbe–microbe interactions, with surfaces acting as a point of anchorage that limits growth and movement in the direction perpendicular to the surface [14, 61–64]. The microenvironments within host that microbes inhabit are often more complex than simple planar surfaces, and thus these models neglect important mechanical contributions [55, 65]. Because of these limitations, questions regarding the impact of the mechanical and spatial characteristics of the host microenvironment on the ecology of polymicrobial communities remain open. For example, it is unclear why the trio of microbes *Pseudomonas aeruginosa*, *Staphylococcus aureus*, and *Candida albicans*, which are difficult to co-culture in the lab [66], are consistently found together in the sputum of patients, particularly those affected by chronic diseases such as cystic fibrosis [67–71]. Do different species in the lung coexist or are they segregated in different regions of the organ?

To investigate the impact of spatial structure on microbial ecology, we compare the spatial organization of polymicrobial communities of *P. aeruginosa*, *S. aureus*, and *C. albicans* in two scenarios: unconfined surfaces and geometrical alveoli mimics. With the latter, we expand the definition of an infection-on-chip, producing microfluidics that do not contain host cells but that reproduce the biochemical properties of the infection site and its key spatial features. This allows us to specifically control and isolate the impact of confinement on ecology, bypassing confounding factors that emerge from microbe–host interactions or from the requirements of host cell culturing. We find that, although *P. aeruginosa* quickly outcompetes the other species on an unlimited surface, alveoli-like confinement profoundly alters ecology, allowing persistent polymicrobial cohabitation. In the geometrical alveoli mimics, we observe the robust emergence of a spatial organization that enables co-existence. Despite growing slower than the other species, *C. albicans* is able to conquer the edges and the closed end of the microcompartments. We demonstrate that this is enabled by a transition to hyphal morphology that inherently focuses directional radial growth. Upon reaching a barrier, *C. albicans* tends to bend and reorient, continuing growth along the edge and displacing other microbes, as reproduced in our purely biomechanical agent-based simulations. Finally, we probe the robustness of the observed spatial organization using a clinically relevant *mexT* mutant of *P. aeruginosa* in which certain virulence factors such as motility and pigments production [72] are overexpressed. Taken together, our results reveal a previously unrecognized role of mechanical forces in shaping infection dynamics and suggest that the microenvironmental structure is a critical determinant of pathogen coexistence, virulence, and treatment outcomes.

## Materials and methods

### Microbial strains, media, and preculture conditions

For *P. aeruginosa*, we used two strains: a PA01 reference strain expressing eCFP [73] from the chromosome and a spontaneous *mexT* frameshift mutant emerging in the same background (and therefore also expressing eCFP). We characterize the strains using whole genome sequencing (MicrobesNG, UK). For *S. aureus* we use a SH1000 strain expressing eGFP from the chromosome [74]. Our reference strain for *C. albicans* is CAF2.1-dTom-NATr that expresses dTomato under the control of the pENO1 enolase promoter [75]. The yeast-locked strain is the WYZ12.2 *hgc1$\Delta $/$\Delta $*, a CAF2.1 derivative from [76]. Prior to any of the experiments, single cultures of *P. aeruginosa*, *S. aureus*, and *C. albicans* are grown overnight in lysogeny broth (0.5% yeast extract, 1% Bacto Tryptone, 0.05% NaCl) in a shaking (220 rpm) incubator at 37$^\circ $C. 1 ml aliquots of each culture were washed twice in phosphate-buffered saline (PBS) (0.8% NaCl, 0.02% KCl, 1.44% Na2HPO4, 0.24% KH2PO4, pH 7.4) with centrifugation (8000xG, 1 min). The single colonies used to inoculate the overnights are streaked on LB plates from frozen stocks on the day before inoculation for *P. aeruginosa* and up to a week before for *S. aureus* and *C. albicans*. Except where otherwise indicated, all of our experiments are carried out in artificial sputum medium (ASM), of which we give a recipe in SI. Our ASM is prepared as in [66] and is therefore a modified version of previously published media [77–79].

### Soft agarose experiments

4 mm thin agarose plates are produced by filling the lids of 3.5 cm petri dishes (Greiner, UK) with 3 ml molten 1.5% agarose solutions in ASM. The plates are seeded with 0.2 $\mu $l mixtures of the washed *P. aeruginosa*, *S. aureus*, and *C. albicans* at various dilution ratios. To prevent agarose marking with the tip of the pipette during seeding, we slowly pipette out the small culture volume until a drop is visible at the end of the tip. The edges of the plates are secured to a custom-made microscope mount with double-sided tape and immediately imaged. This is carried out using a Nikon 10$\times $ Plan Apo air objective with a numeric aperture of 0.45 on a Nikon Ti-E microscope using Nikon Perfect Focus System. A total of 280 images per colony were collected using a Teledyne FLIR BFS-U3-70S7M-C with a 7.1 MP Sony IMX428 monochrome image sensor. All images are captured at 3208$\times $2200 pixels, with a resolution of 0.43 $\mu $m/pixel. Each field of view (FOV) is imaged seven times, of which one in brightfield and three in three fluorescence channels, each imaged at two different exposure time and gain combinations: 0.5 s, 20 gain and 0.2 s, 10 gain for dTomato; 0.2 s, 32 gain and 0.005 s, 32 gain for eGFP; 0.06 s, 32 gain and 0.005 s and 32 gain for eCFP. Illumination is provided by color Light Emitting Diodes: red for brightfield (Lumileds Luxeon Z LXZ1-PD01), indigo for eCFP (Lumileds Luxeon UV LHUV-0425), blue for eGFP (Lumileds Luxeon Z LXZ1-PB01), and lime for dTomato (Lumileds Luxeon Z LXZ1-PX01). For fluorescence imaging we use Semrock optical filter sets (IDEX Health and Science, USA), CFP-2432C for eCFP, GFP-3035D for eGFP, and TxRed-4040C for dTomato. After imaging, which typically took 2 h, positions of the colonies on the plates are recorded and the plates are temporarily sealed with a second lid glued with Covergrip coverslip sealant (Biotium, USA) to maintain moisture. The plates are transferred to a static 37$^\circ $C incubator for 24 h and imaged again.

### Microfluidic lung mimic production

Microfluidic master molds are produced in-house using SU8 (Kayaku AM, USA) soft-lithography following the manufacturer’s guidelines. A first thin layer of SU8-2010 is deposited on a 10.16 cm (4 inches) silicon wafer (PI-KEM, UK), exposed to UV light in a MicroWriter ML3 Pro (Durham Magneto Optics, UK) and baked to obtain a final thickness of $\sim $8 $\mu $m (Fig. 2C). A second layer of SU8-2075 is then deposited on the mold, exposed to UV light, baked and washed with SU8 developer (Kayaku AM, USA) to obtain the main trench (Fig. 2A), with a height of 0.3 mm. The designs are inspired by the family machine [80]. The molds are then used to fabricate microfluidic chips using degassed PDMS at a 1:10 ratio between curing agent and elastomer. Curing of the PDMS was performed in a 60$^\circ $C oven. Before use, chips are released from the mold using a scalpel, inlets and outlets are punched using a biopsy puncher with 0.77 mm internal diameter (World Precision Instruments-Europe, UK), and the chip is plasma bonded to a 0.145 mm thick glass coverslip (VWR, UK) and baked at 60$^\circ $C for at least 10 min. For the microfabrication of a mother machine that could house *C. albicans*, we use the same procedure, but lower the height of the first SU8 layer to 6 $\mu $m.

**Figure 1:**
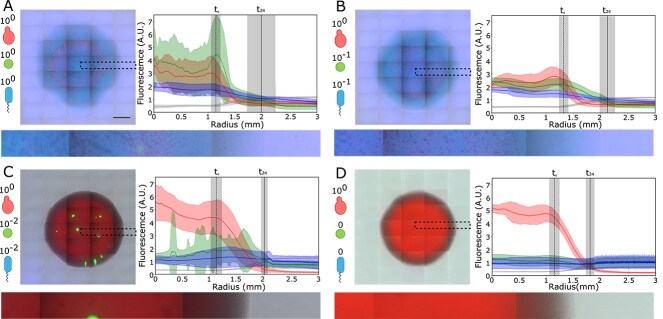
Quantification of the growth of a PA-SA-CA community on an unlimited surface. (A) PA-SA-CA polymicrobial community seeded at a 1:1:1 ratio on soft agar. Left: example colony after 24 h growth at 37$^{\circ }$C. Bottom: zoom-in of the highlighted area. Right: average intensity profiles for the red, green, blue (fluorescence) and gray (brightfield) channels. Gray reports brightfield intensity and is a proxy for total colony size. To obtain the traces, colonies are split in 360 sections, each 1$^{\circ }$ large. Radii between the centroid and the edges of the image are drawn and averaged. The shaded areas show the standard deviation of all radii across five colonies. The vertical black lines indicate the average radius of the colony at the start and after 24 h. The shaded area shows the standard deviation. Scale bar = 1 mm. (B) PA-SA-CA polymicrobial community seeded at a 0.1:0.1:1 ratio. Averages and standard deviations of three colonies. (C) PA-SA-CA polymicrobial community seeded at a 0.01:0.01:1 ratio. Averages and standard deviations of three colonies. (D) Growth of a colony of *C. albicans* in isolation. Averages and standard deviations of three colonies. Epifluorescence microscopy, stitched FOVs. The bottom strips are zoom-ins of the areas highlighted by the dotted lines. Channel-separated, grayscale versions of this figure are given at the end of SI.

**Figure 2:**
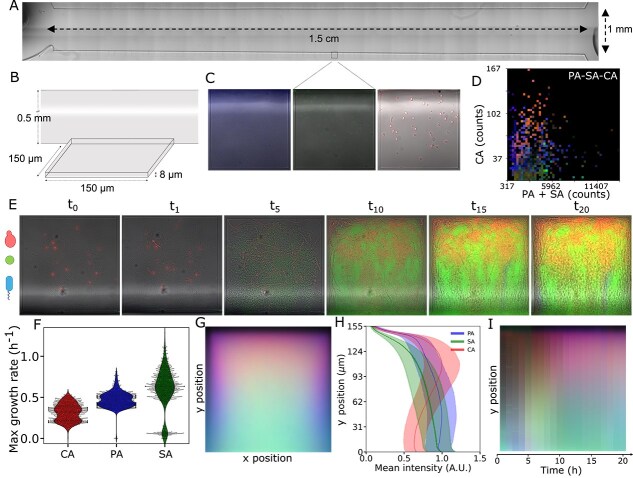
Quantification of the growth dynamics of a PA-SA-CA community in spatially structured environments. (A) Architecture and dimensions of the microfluidic chip. (B) Zoom-in to the dimensions of the quasi-2D alveoli-inspired geometries. (C) *Pseudomonas aeruginosa* (left, blue), *S. aureus* (center, green), and *C. albicans* (right, red) at the seeding. They express eCFP, eGFP, and dTomato, respectively. (D) Mean fluorescence for dTomato, eGFP, and eCFP expressed as RGB color in the distal quarter (closed end) of the microchambers after 20 h, plotted as a function of the initial seeding conditions (number of yeast cells against number of bacterial cells). (E) example time traces from different experiments of the PA-SA-CA community growing in the alveoli-inspired geometries. (F) Maximum growth rates extracted from the time course of the different fluorescence channels. $P$ values obtained with Welch’s t-test are all $< 1e-47$. (G) Average behavior at 20 h, obtained by summing up all of the boxes. (H) Average and standard deviation of the fluorescence intensity of the three fluorescence channels, proxy for PA, SA, and CA abundance, along the vertical axis of (G). (I) Kymograph showing the progression of the average behavior over 20 h of observation. Each band is obtained by averaging the mean image (G) along the $x$-axis. In all cases, the side length of the square alveolar geometries is 150 $\mu $m. In all cases, the figures are generated from data from nine independent experiments with 100 replicates (boxes) each. Channel-separated, grayscale versions of this figure are given at the end of SI.

### Lung-mimic experiments preparation

Microfluidic channels are loaded with microbes mixtures obtained from the washed overnight cultures and inspected using microscopy. To carry out the experiment, inlets and outlets of the microfluidic chips are connected to Tygon tubing (Cole Parmer; 0.020” $\times $ 0.060” outside diameter), using 90$^\circ $ bent connectors (Intertronics, UK; stainless steel, 0.89-0.58 OD-ID, 20 gauge) as before [81]. A 20 $\mu $/min flow of ASM medium is prompted by a syringe pump (kdScientific, USA), loaded with a 60 ml plastic syringe (BD plastipak, UK), connected to the tubing via blunt needles (Intertronics, UK; stainless steel, straight blunt, 1/2”, 23 gauge).

### Lung-mimic experiments

Experiments are carried out at 37$^\circ $C by housing the microfluidic chip in custom-built heating elements that are fitted to the automated xy stage of a Nikon Eclipse Ti-E. Before starting the experiment, and after loading, an arbitrarily fast flow pulse of fresh ASM medium is delivered to the main trench of the chip to remove microbes that had not entered in the alveoli-mimicking boxes. Imaging is automatically carried out in two phases. In the first phase, we imaged the 100 boxes on one side of the channel using a Nikon 40$\times $ CFI Plan Fluor air objective with a numeric aperture of 0.75. Each box is imaged 19 times, once in brightfield and 18 times in fluorescence using the same LEDs and filters given in the *Soft agarose experiments* section. Each fluorescence channel is imaged at maximum gain at three different z offsets, with two exposure times: 0.4 and 0.1 s for dTomato; 0.05 and 0.003 s for eGFP; 0.5 and 0.05 s for eCFP. In the second phase, we image the whole channel using the 10x air objective described in the *Soft agarose experiments* section. At this stage, we collect 266 images per channel for a total of 38 FOVs, each imaged seven times: once in brightfield and twice per each fluorescence channel, using 32 gain and different exposure times: 0.5 and 0.2 s for dTomato; 0.05 and 0.005 s for eGFP; 0.06 and 0.005 s for eCFP. The two phases combined typically take 54 min of microscope time and are automatically repeated every hour.

### Mother machine experiments

To evaluate the correspondence between biomass and fluorescence along various growth stages in the three strains we use mother machines. This allows us to track fluorescence in single cells while extracting their biomass based on their cross-sectional area. Because the cells are confined within pistons, the cross-sectional area provides a good approximation of the volume, as cells cannot stack on top of each other and are uniformly oriented. This allows us to characterize fluorescence with the same system used for data collection. In addition, it allows us to bypass the issues associated with bulk measurements of microbes that change morphology, that form clumps and biofilms, and that secrete fluorescent pigments (SI Fig. 4D). In particular, we use two mother machines: one with pistons with width and height below 2 $\mu $m [81] for *P. aeruginosa* and *S. aureus* and one with pistons with width and height below 6 $\mu $m for *C. albicans*. Before loading the cells in the pistons, the channels are incubated overnight at 37$^\circ $C with a 2% solution of bovine serum albumin. Loading is carried out from 50x concentrated LB overnight cultures, double-washed in PBS in all cases. For *P. aeruginosa* and *S. aureus* loading occurred spontaneously, whereas for *C. albicans* we enhance it by spinning the mother machine at 1000 rpm in a spin coater for 1 min. Once loading is satisfactory, the inlet and outlet of the mother machine are attached to a syringe pump, as described above, using a 5 ml syringe (BD plastipak, UK). The chip is heated to 37$^\circ $C and ASM is flowed in at 5 $\mu $l/min. Further explanations on the extraction of fluorescence and biomass are given in SI.

### Data analysis

Details on data analysis and simulations are given in SI.

## Results

### *P. aeruginosa* quickly outcompetes *S. aureus* and *C. albicans* on a surface

We consider a lung-relevant pathogenic polymicrobial community of *P. aeruginosa* (PA), *S. aureus* (SA), and *C. albicans* (CA) on the simplest and best studied [23] example of a structured environment: a soft agarose surface. The species in our community, express different fluorescent proteins that allow their tracking in epifluorescence microscopy: eCFP for PA, eGFP for SA, and dTomato for CA. The strains are PA01 for PA, SH1000 for SA, and CAF2.1 for CA. To recapitulate aspects of lung biochemistry, plates are composed of ASM [66]. Using a micropipette, we seed small aliquots of cells from the three species mixed in different ratios (1:1:1, 0.1:0.1:1, 0.01:0.01:1) and observe their growth at 37$^\circ $C after 24 h. When the inoculum contained the same number of cells of the three species, despite the much lower cell size and starting biomass, *P. aeruginosa* dominated the mixed colony after 24 h. The colonies nearly doubled their radii over the time interval. Whereas *C. albicans* and *S. aureus* show limited growth and remain confined to their seeding spots, all of the radius gained by the colony over the 24 h is due to *P. aeruginosa* growth (Fig. 1A). This is consistent with reports indicating that motility is a major driver of growth on surfaces [11, 82–84]. To understand the extent of such advantage, we repeat the experiment at two more PA:SA:CA seeding ratios: 0.1:0.1:1 and 0.01:0.01:1. Reducing the PA titer by 10 times does not significantly affect the colony growth phenotype observed, with PA still dominating (Fig. 1B). The phenotype switches to CA dominance when its initial titer is 100 times higher (Fig. 1). The colony radius also doubles over 24 h, confirming competitive inhibition between PA, CA, and SA, as previously observed [66].

### Spatial organization emerges within 10 h in a lung mimic

Agar surfaces, such as the one we used, model situations in which space is unlimited in the planar surface and are therefore best suited to study unstructured environments such as the upper airways. The environment of the lower airways is significantly more structured, with alveolar volumes of the order of millions of cubic micrometers, which, if approximated to spheres, would measure 100 to 250 $\mu $m in diameter [85, 86]. To model the effects that spatial limits have on the ecology of our pathogenic polymicrobial community, we present a microfluidic model inspired by the geometrical constraints of the distal lung. Our design is similar to the family machine [80], and encompasses a central channel 1.5 cm long, 1 mm wide, 0.3 mm tall, lined on each side by 100 quasi-2D square geometries with a side length of 150 $\mu $m and a thickness of 8 $\mu $m (Fig. 2A and B). All measures are modeled on those of the distal lung with the exception of the thickness of the alveolar space, which we chose to limit to 8 $\mu $m. By choosing this thickness we are able to observe and quantify the contents of a single slice of the space that geometrically mimics alveoli without the need to use confocal microscopy, improving our imaging throughput. The thickness of these alveoli-inspired geometries allows the stacking of multiple *P. aeruginosa* and *S. aureus* cells, whereas *C. albicans* cells are limited to one (Fig. 1C, SI Fig. 1). In larger spaces with side length of 500 $\mu $m, a slowdown of growth emerges toward the closed end, indicating the development of nutritional gradients (SI Fig. 2A). Cells are loaded in the alveoli-inspired geometries at different species titers (Fig. 2D) and continuously perfused with fresh ASM for up to 20 h while we perform timelapse microscopy. The observation window is limited to this interval because at later stages robust biofilm growth in the main channel starts influencing cell behavior in the alveoli-inspired geometries (SI Fig. 3). Across nine independent experiments (Fig. 2D) and 900 alveoli-inspired geometries, the robust emergence of spatial organization is observed (Fig. 2E and G) with a tendency of *C. albicans* to localize toward the closed end and the edges (SI Video 1). This displaces *P. aeruginosa* and *S. aureus*, which tend to localize toward the center and the entrance of the box. The displacement is most evident in *S. aureus* (Fig. 2H). This spatial organization emerges in <10 h and remains stable throughout the course of the observations (Fig. 2I). As expected, the outcome has a certain dependence on the seeding titer of the species, with high *C. albicans*-to-bacteria ratios leading to dominance by the fungus and vice versa (Fig. 2D). To ensure that fluorescence measurements in the geometrical alveoli mimics are a good proxy for the biomass of each species, we carry out control experiments inside species-specific mother machines (SI Fig. 4). These allow us to simultaneously extract biomass from the z-limited cross-area of the cells and their fluorescence across different growth stages. Because the two quantities are in good agreement, we utilize the time traces of each fluorescence channel to extract the growth rate of each species in the polymicrobial context within the alveoli-inspired geometries. Despite the observed sharing of space and the robust tendency of *C. albicans* to conquer the closed end of the alveoli-inspired geometries, we find significantly different growth rates, with the fungus growing the slowest (SI Fig. 5).

### Spatial organization is robust to the presence of a clinically relevant *P. aeruginosa mexT* mutant

**Figure 3:**
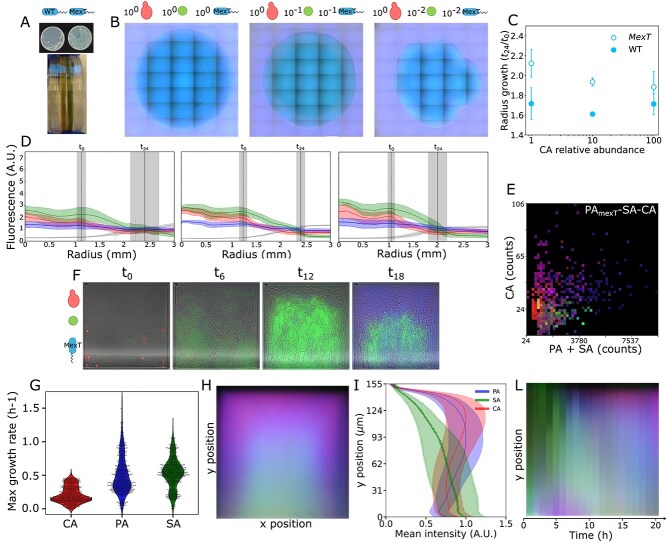
Test of the robustness of the spatial organization of a PA-SA-CA community in which *P. aeruginosa* carries a clinically relevant *mexT*-inactivating mutation. (A) The *mexT* mutant shows a characteristically enlarged colony size (top) and secretes large quantities of piocyanin (bottom). (B) PA$_{\mathit{mexT}}$-SA-CA polymicrobial community seeded at various ratios (left 1:1:1, center 0.1:0.1:1, right 0.01:0.01:1). The *P. aeruginosa* strain expresses the mutated *mexT*. Scale bar = 1 mm. (C) Comparison of radius growth between PA-SA-CA communities with a reference strain of *P. aeruginosa* (filled circles) or the *mexT* mutant (empty circles). The error bar shows the standard deviation between repeats (six colonies for a PA-SA-CA initial titer of 1:1:1, three colonies for 0.1:0.1:1, and six colonies for 0.01:0.01:1. $P$ values for 1, 10, and 100 CA relative abundance are: 0.004, 0.004, and 0.15, respectively. (D) Average intensity profiles for red, green, blue (fluorescence) and gray (brightfield) channels. Gray indicates brightfield intensity as a proxy for total colony size. Traces are obtained as the average of 360 radii, one every angular degree of the colony. The shaded areas show the standard deviation. The vertical black lines indicate the average radius of the colony at the start and after 24 h. The shaded area shows the standard deviation. Left: PA-SA-CA ratios 1:1:1, center (0.1:0.1:1), right (0.01:0.01:1). (E) Mean fluorescence for dTomato, eGFP, and eCFP expressed as RGB color in the distal quarter (closed end) of the microchambers after 20 h, plotted as a function of the initial seeding conditions (number of yeast cells against number of bacterial cells). (F) example time trace of the three-species polymicrobial community containing the *mexT* mutant in the geometrical alveoli mimics. (G) Growth rates extracted from the timelines of the fluorescence channels. (H) Average behavior at 20 h, obtained by summing up all of the boxes. (I) Average and standard deviation of the fluorescence intensity of the three fluorescence channels, proxy for PA, SA, and CA abundance, along the vertical axis of (H). (L) Kymograph showing the progression of the average behavior over 20 h of observation. Each band is obtained by averaging the mean image (H) along the $x$-axis. In all cases, the side length of the square alveolar geometries is 150 $\mu $m and the figures are generated from data from six independent experiments with 100 replicates (boxes) each. Channel-separated, grayscale versions of this figure are given at the end of SI.

As a first test of the robustness of the emerging spatial organization against changes in the virulence of its members, we replaced the *P. aeruginosa* PA01 reference strain with a *mexT* frameshift mutant spontaneously emerged in the same background. Inactivation of *mexT* is a common feature in clinical isolates, leading to increased expression of virulence factors such as motility and pigments secretion [72]. The *mexT* mutant tends to form large rough colonies, which express significant amounts of a green pigment called pyocyanin [87] (Fig. 3A). Microscopy of the colonies on ASM agarose surfaces shows increased fitness of the mutant compared with the already dominant reference strain (Fig. 3B, C and D). *mexT* polymicrobial colonies grow larger (Fig. 3C), with *C. albicans* outcompeted even when 100 times more abundant than *P. aeruginosa* and *S. aureus* at the seeding (Fig. 3B and D). In the geometrical alveoli mimics with the *mexT* mutant, seeding communities at different species titers (Fig. 3E), again, shows that spatial organization emerges robustly across six independent experiments and 600 alveoli (Fig. 3F and H). In this case, the exclusion of *P. aeruginosa* from the closed end is less severe, but more severe for *S. aureus* (Fig. 3I). Although the mutant has a slightly higher growth rate than the reference strain (Fig. 3G and SI Fig. 4C), spatial organization still emerges within 10 h (Fig. 3L, SI video 2) and at the same seeding titers (Fig. 3E).

### Species composition influences spatial organization

**Figure 4:**
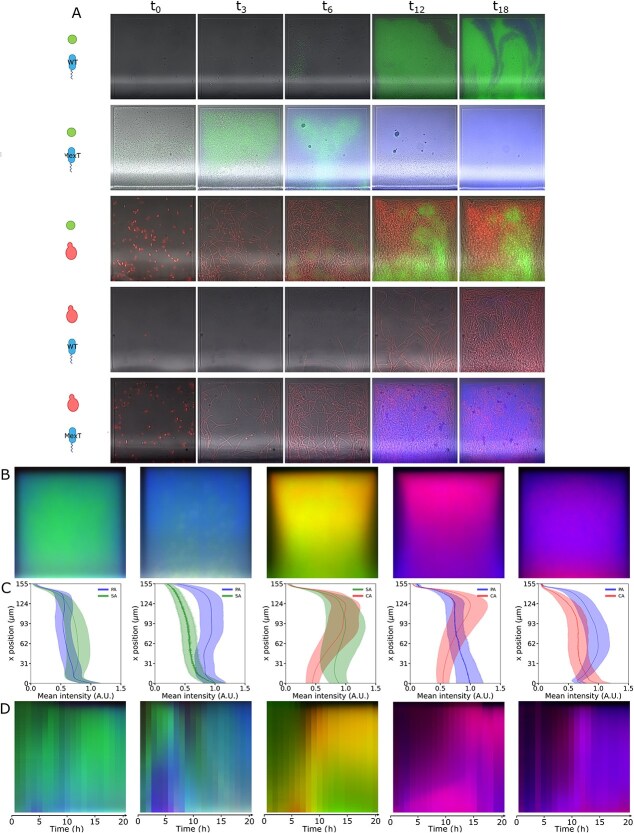
Analysis of the role of each species in the determination of spatial organization. (A) Example time series of PA-SA, PA$_{mexT}$-SA, SA-CA, PA-CA, and PA$_{mexT}$-CA communities. (B) Average behavior at 20 h, obtained by summing up all of the boxes (three experiments, 300 boxes each). Spatial organization emerges also in the absence of SA or PA, but not if PA has a mutated *mexT*. (C) Average and standard deviation of the fluorescence intensity of the three fluorescence channels, proxy for PA, SA, and CA abundance, along the vertical axis of (B). (D) Kymograph showing the progression of the average behavior over 20 h of observation. Each band is obtained by averaging the mean image (B) along the $x$-axis. In all cases, the side length of the square alveolar geometries is 150 $\mu $m and the figures are generated from data from at least three independent experiments with 100 replicates (boxes) each. Channel-separated, grayscale versions of this figure are given at the end of SI.

Having characterized the behavior of a three-species community, we investigate the contributions of single members by studying pair interactions. When grown in the absence of *C. albicans*, *S. aureus*, and the *P. aeruginosa* reference strain tend to share the niche (Fig. 4, SI video 3). The *mexT* mutation alters this balance, resulting in a characteristic succession pattern in which *S. aureus* initially grows robustly to then be removed from the box by *P. aeruginosa* that systematically conquers the edges (Fig. 4A, second row, SI Video 4). In the presence of *C. albicans*, the absence of either *P. aeruginosa* (SI Video 5) or *S. aureus* (SI Video 6) shows little impact on spatial organization, with the yeast preferentially occupying the edges and the closed end of the alveoli-inspired geometries after 8–12 h as in the three-species scenario (Fig. 4). Similar to the *P. aeruginosa*–*S. aureus* pair, *mexT* shifts the balance toward *P. aeruginosa* (Fig. 4, SI Video 7).

### *C. albicans* conquers the closed end of confinements by growing radially with hyphae

**Figure 5:**
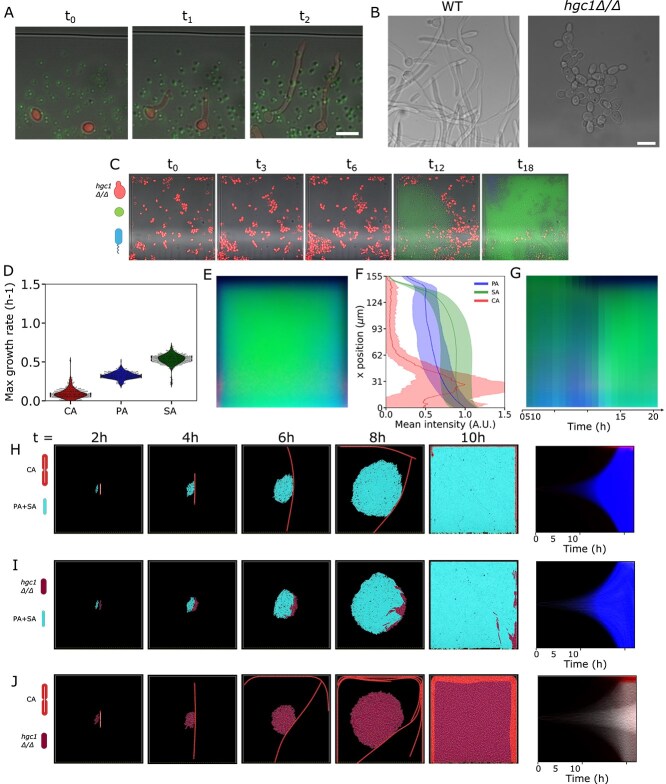
Analysis of the role of hyphal growth in the determination of spatial organization and coexistence. (A) Representative transition from yeast to hyphal form of *C. albicans* within geometrical alveoli mimics. (B) The yeast-locked mutant *hgc1*$\Delta $:$\Delta $ does not grow hyphae in our conditions. (C) Example time lapse of the *hgc1*$\Delta $:$\Delta $ growing in the presence of *P. aeruginosa* and *S. aureus*. *Candida albicans* is segmented from brightfield and false colored as explained in the methods section. (D) Growth rate of the three species grown in the geometrical alveoli mimics. (E) Average behavior at 20 h, obtained by summing up all of the boxes (five experiments, 500 boxes each). (F) Average and standard deviation of the fluorescence intensity of the three fluorescence channels, proxy for PA, SA, and CA abundance, along the vertical axis of (E). (G) Kymograph showing the progression of the average behavior over 20 h of observation. Each band is obtained by averaging the mean image (E) along the $x$-axis. (H–J) 2D agent simulations initialized with two cells at the center of the trap showing the following: (H) that hyphae-competent *C. albicans* positions itself at the edges; (I) that the yeast-locked mutant *hgc1*$\Delta $:$\Delta $ does not enable spatial organization, even when it grows at the same growth rate of the reference strain; (J) that a hyphae-competent species occupies the edges of confinement in the presence of a species that grows at the same rate, but that cannot form hyphae. At the end of each row, kymographs summarizing the dynamic progression of the simulations across at least nine repeats are given. In the kymographs, the *hgc1*$\Delta $:$\Delta $ is given in white. Scale bars = 10 $\mu $m. In all cases, the side length of the square alveolar geometries is 150 $\mu $m. Channel-separated, grayscale versions of this figure are given at the end of SI.

Our results show that *C. albicans* tends to occupy the edges of confinements and the closed end of our alveoli-inspired geometries. This could be the main driver of spatial organization. We therefore seek to investigate how the slowest growing species can have such a dominant role in the ecology of a pathogenic polymicrobial community. Although *C. albicans*’s biomass grows overall more slowly in the alveoli-inspired geometries, its transition to hyphae in ASM focuses all growth in radial directions by virtue of the long asymmetric morphology of these structures (Fig. 5A). Although growth on a surface is known to stimulate hyphal transition, the ASM used here possesses all the necessary and sufficient conditions to induce hyphal morphogenesis (SI Fig. 6). In confinement, this allows it to rapidly reach the edges of the alveoli-inspired geometries and align along the walls. To test whether hyphal growth is necessary for spatial organization, we repeated polymicrobial experiments using a yeast-locked *C. albicans* strain that does not form hyphae, as it lacks the HGC1 gene, encoding a cyclin-related protein necessary for hyphal transition in ASM (Fig. 5B). This abolishes the spatial organization observed with the hyphae-competent strain (Fig. 5C, 5D, 5F and 5G, SI Video 8). However, the *hgc1$\Delta $:$\Delta $* strain has a slower growth rate than the reference strain (SI Fig. 5).

To further test whether the radial growth conferred to *C. albicans* by its hyphae determines spatial organization, we simulate a mutant with an identical growth rate *in silico*, using 2D agent-based simulations. A 2D alveoli-inspired geometry is populated by two species with growth rates and cell sizes that match those observed in experiments for the reference strain *C. albicans* and bacteria (grouping together *P. aeruginosa* and *S. aureus*, see *Methods* section). A single cell of each is initialized in the middle of the geometrical alveoli mimic. Strikingly, the hyphal *C. albicans* reside at the edges of the geometrical alveoli mimic, reproducing the spatial organization observed in experiments (Fig. 5H, SI Video 9). To directly test the role of radial *C. albicans* growth, we switch the reference *C. albicans* with a *hgc1$\Delta $:$\Delta $*-like strain that does not form elongated cells but has the same growth rate (Fig. 5I). In this case, the species mix without producing spatial organization (SI Video 10). To further confirm this is due to hyphae and not to the different single cell sizes, we simulate growth of hyphal *C. albicans* and yeast-locked mutant within the same niche at identical growth rate and again observe the localization of the cells that form hyphae at the periphery (Fig. 5J, SI Video 11).

Our simulations (Fig. 5) are initialized with both species starting with the same number of cells (one) at the center of the niche; however relative numbers and starting positions are expected to impact the efficacy of radial growth for establishing and maintaining spatial structure. Simulations that start with large numbers of CA and bacterial cells homogeneously spread throughout the niche exhibit less pronounced spatial structure (SI Fig. 7). These predict that spatial organization emerges at intermediate times before eventual bacterial dominance. A certain dependence on relative numbers is observed also in the experiments (Fig. 2D and E). These results demonstrate that spatial structure within the geometrical alveoli mimics is driven by the asymmetrical growth of *C. albicans* hyphae, which, by concentrating in one direction, allows them to extend to the walls and corners of the mimic, though initial cell position and numbers could negate this advantage. Because the simulations only consider growth rates and mechanical interactions, they suggest that *C. albicans*’ dimorphism is sufficient to induce spatial organization. Although other mechanical and biochemical interactions are possible, they are not necessary to recapitulate the observed spatial organization and coexistence.

## Discussion

By comparing agarose surfaces and confining microfluidic alveoli-inspired geometries, we have shown that spatially structured niches can alter ecological outcomes in pathogenic microbes. The quasi-2D confinements reveal that, within 10 h after seeding, a polymicrobial community of *P. aeruginosa*, *S. aureus*, and *C. albicans* acquires and maintains a characteristic spatial organization that is not achieved on unconfined planar surfaces. The pivotal factor in this behavior is the capability of *C. albicans* to line the edges of the confined space with its hyphae, limiting the presence of the other members of the community. This strategy allows the fungus to survive in the niche despite its slow growth rate, coexisting with the faster growing *S. aureus* and *P. aeruginosa*. In contrast, in the absence of spatial limits on a planar surface, *P. aeruginosa* quickly outcompetes *C. albicans*, even when starting from a 100-fold numerical disadvantage. Like in real alveoli, the edges of the alveoli-inspired geometries mediate the contacts between microbes and substrate. In real alveoli, this contact is negotiated by the respiratory epithelium. Therefore, due to its tendency to line confined spaces, *C. albicans* may mediate most of the physical contact between the host and the polymicrobial community *in vivo*. This could have repercussions on treatment design, e.g. because targeting *C. albicans* might lead to its replacement with more pathogenic microbes. Instead, strategies that blunt *C. albicans*’ toxicity without removing it from the niche may protect the host from further damage.

Our quasi-2D models are inspired by environments that would normally be 3D and although the model enables us to specifically study the impact of edges and geometry, other factors such as nutrients gradients might become more important in 3D situations, where cells in the bulk might have more difficult access to these chemicals. Many other differences exist in comparison with pulmonary alveoli, including the fact that these do not normally tend to be flooded before infection. As in the case of edema, flooding can occur as a response to infection and tissue damage. Our model is therefore better suited to describe these conditions rather than the colonization of a sterile alveolar space. In the latter, microbes would start colonization already close to the edges and dynamics might be different. Although this will require further research, we expect the mechanisms observed in this work to still be relevant in these conditions, with hyphae still able to displace other microbes. Indeed, in the case of a thin liquid layer, surface tension would narrow down the range of possible orientations of hyphal tip growth, limiting it to those pointing toward the surface or parallel to it.

The robustness of the observed spatial organization and coexistence was tested with a *P. aeruginosa mexT* mutant, an adaptation that is frequently found in clinical isolates [72]. *mexT* is a transcription regulator that controls the expression of >40 genes [88]. Its deletion increases virulence by upregulating motility and the production of secreted virulence factors, such as pyocyanin [87]. Although in the three-species experiments considered here, the mutant does not qualitatively alter the overall spatial organization trend, it does show increased ecological competitiveness. This is evident from a comparison between Fig. 2G and Fig. 3H with the mutant tending to a distribution that is skewed toward the edges of the microenvironments. Its increased ecological competitiveness manifests itself fully in two-species infections, where the *mexT* mutant is able to completely eradicate *S. aureus* and limit *C. albicans*’ number (Fig. 4A). Because its growth rate is similar to that of the reference strain (SI Fig. 5, SI Fig. 4C), and those of *S. aureus* and *C. albicans* are not particularly affected by its presence, we hypothesize that its advantage is due to increased motility. This could allow the bacterium to make its way around *C. albicans*’ biomass and dislodge *S. aureus* from crevices, pushing it away through growth. Although on the unconfined surface we found indications of competitive inhibition, likely due to the dynamics of nutrients usage, we did not observe them in the microenvironments that are continuously perfused. Other mutations that upregulate contact-dependent and independent toxin production both in the lung context and beyond might alter the balance we have observed.

As a mechanism for the emergence of spatial organization, we hypothesized that hyphae allow *C. albicans* to concentrate its growth in a single direction, enabling it to reach the edges of the confinement before being removed by the faster growing species. *In silico* experiments of single cells placed in direct competition confirm this view. We further tested this hypothesis with a yeast-locked mutant that does not form hyphae, finding that it does not give rise to spatial organization or persist in the alveoli-inspired geometries. However, the mutant has a slower growth rate than the reference strain, and thus we repeated the experiment *in silico*, this time matching the growth rates. The simulations confirmed our hypothesis, indicating that an elongated morphology in a slower growing species is sufficient to offset the ecological outcome in a confinement.

Taken together, our findings demonstrate that traits controlled at the level of phenotype, such as *C. albicans*’ morphological changes and *P. aeruginosa*’s virulence, can drastically change ecological results when observed in realistic settings to the point of outweighing the growth rate. Because such evolutionary adaptations are prevalent among microbes and microscale spatial structure is widespread across different environments, we expect many other examples to exist in nature.

## Supplementary Material

SI_compressed_final_wraf279

SI_video1_wraf279

SI_video2_wraf279

SI_video3_wraf279

SI_video4_wraf279

SI_video5_wraf279

SI_video6_wraf279

SI_video7_wraf279

SI_video8_wraf279

SI_video9_wraf279

SI_video10_wraf279

SI_video11_wraf279

## Data Availability

Data are available at 10.5281/zenodo.15005092. Scripts used for analysis are available at https://github.com/mlaenoc/alveoli-code. Code for the simulations is available at https://github.com/roryclaydon1994/BiofilmDES.
