# Supplementary material for: Hyphal growth determines spatial organization and coexistence in a pathogenic polymicrobial community in a spatially structured environment
Source: ISME J. 2025 Dec 18;19(1):wraf279. doi: 10.1093/ismejo/wraf279 (PMC12753314; doi:10.1093/ismejo/wraf279)
Supplement: SI_compressed_final_wraf279 [file si_compressed_final_wraf279.pdf]

Hyphal growth enables spatial organization and coexistence in a pathogenic polymicrobial community in a spatially structured environment

Leonardo Mancini[1,2+], Laila Saliekh[3], Rory Claydon[3], Jurij Kotar[1], Eva Bernadett Benyei[2], Carol A Munro[4], Tyler N Shendruk[3], Aidan Brown[3], Martin Welch[2*], and Pietro Cicuta[1*]

[1]Department of Physics, Cavendish Laboratory, University of Cambridge, J.J. Thomson Avenue, Cambridge, Cambridgeshire, CB3 0HE, UK, lm653@cam.ac.uk
[2]Department of Biochemistry, University of Cambridge, Cambridge, UK
[3]School of Physics and Astronomy, The University of Edinburgh, Edinburgh, UK
[4]Institute of Medical Sciences, University of Aberdeen, Aberdeen, UK
[+]Corresponding author
[*]Equal contribution

Supplementary Methods

ASM preparation

Our ASM is prepared as follows: (saturating amount (< 5 g/L) of mucin from porcine stomach type-II dissolved in PBS, saturating amount (< 4 g/L) of fish sperm DNA, 1.3 mM NaH2PO4, 1.25 mM Na2HPO4, 0.348 mM KNO3, 0.271 mM K2SO4, 2.28 mM NH4Cl, 14.94 mM KCl, 51.85 mM NaCl, 10 mM MOPS, 1.45 mM Serine, 1.55 mM Glutamic acid, 1.66 mM Proline, 1.2 mM Glycine, 1.78 mM Alanine, 1.12 mM Valine, 0.63 mM Methionine, 1.12 mM Isoleucine, 1.61 mM Leucine, 0.68 mM Ornitine, 2.13 mM Lysine, 0.31 mM Arginine, 0.01 mM Tryptophan, 0.83 mM Aspartic acid, 0.8 mM Tyrosine, 1.07 mM Threonine, 0.16 mM Cysteine, 0.53 mM Phenylalanine, 0.52 mM Histidine, 3 mM Glucose, 9.3 mM L-lactic acid, 1.75 mM CaCl2, 0.6 mM MgCl2, 0.0036 mM FeSO4, 0.3 mM N-acetylglucosamine, 5 ml Egg yolk emulsion, pH 6.8, filter sterilised).

Plate reader experiments

Plate reader experiments were carried out in a Spectrostar Omega microplate reader (BMG, Germany). The strains used were: *P. aeruginosa* PA01 (PA) and PA01 with a spontenously inactivated *mexT* (PA *mexT*), *S. aureus* SH1000 (SA) and SH1000-EGFP (SA EGFP), *C. albicans* SC5314 (CA) and CAF2.1-dTomato (CA dTOM). *mexT* inactivation emerged spontaneously in both reference and fluorescent strain of *P. aeruginosa*, they were caused by frameshift mutations and confirmed by sequencing. Cultures were grown to stationary phase in LB (bacteria) or YPD (yeast) and inoculated in fresh LB or YPD in 96-well plates at a 1:100 dilution. Optical density and fluorescence readings were performed at 37○C at intervals of 7.5 minutes. The intervals included 3 minutes of shaking in double orbital mode at 700 rpm. Fluorescence was measured using 450ex/520em (blue/ECFP), 485ex/520em (green/EGFP), and 530ex/645em (red/dTomato) filters. To compare growth curves, lag times were uniformed by aligning optical density curves to OD600 = 0.5.

Data analysis

The code used for image analysis and statistics is available at: 10.5281/zenodo.15005092.

Image correction and preprocessing

Image processing is performed in Python, Fiji [1], and ilastik [2]. Brightfield and fluorescence images from ASM-agarose surfaces (Fig. 1) are normalized by the illumination profiles of the respective light sources before stitching. The profiles are obtained by averaging the signal from at least 15 FOVs captured on a coverslip without any objects in focus. In all cases, stitching is performed using a custom-made Python script with the exception of Fig. 2A, for which we use the Grid/Collection stitching plugin by [3] in Fiji. Because the fluorescence change is relatively small at the center of the images where the alveoli-mimicking boxes are captured, we choose not to perform such illumination profile correction in any of the other microscopy images presented in the text. The FOV of the camera with the 40x air objective is 382x262 μm and therefore we are able to image two boxes at a time. To extract single boxes, we

use a Python-enhanced manual approach in which, for each image, frames of 1400x1400 pixels are superimposed to the brightfield images and their position manually fine-tuned to match the microfluidic boxes. This is done for the 100 positions of the boxes. Choosing a frame size that is slightly larger than the box, coupled with the robustness of the stage control allows us to bypass image registration. During experiment setup, we also strived to minimize tilt along the channel, which allows us to neglect rotational adjustments at the time of analysis. RGB images of single FOVs are generated in Fiji by merging the original 16-bit images in the gray, red, green, and blue channels. We take fluorescence images at three offsets in the box: top and bottom surfaces and middle. For the presentation of single FOVs, we use the bottom offset (glass surface) as this captured most of the *P. aeruginosa* and *S. aureus* cells in the early time points and the central one for the fluorescence channel of *C. albicans.* The final images are exported as 24-bit RGB-color images. The average images (Fig. 2F, 3F, 4B, 5E) are obtained by adding together in the RGB channels the fluorescence images of 100 boxes per experiment per time point. To capture the total behavior in the box, we use the central offsets for all channels. The values are normalized by the maximum value found for the specific fluorescence channel across the entire time series. In our large dataset (>1.5M tiff images across 50 experiments for the microfluidic experiments alone), we capture rare events in which the microscope-camera combination fails to acquire single frames. When an image at a given offset is found missing or corrupted, we replace it with the closest offset at the same position. When none of the alternative offsets are available, we replace the image with its closest available preceding time point. Kymographs are obtained by averaging the sums of 20 consecutive time points along the image's x-axis. To enhance visibility and produce square kymographs, each time point is presented as a 70-pixel wide column. Before plotting the fluorescence profiles (Fig. 2G, 3H, 4C and 5F) along the y-axis of the summed images, we apply the illumination profile correction explained above. Because the *hgc1*Δ:Δ *C. albicans* strain shows a reduced growth rate, we choose not to burden it further with the expression of dTomato. Instead, segmentation is carried out on the brightfield images using ilastik [2]. Pixels belonging to the cells (foreground) and the background are assigned arbitrary fluorescence values of 20000 and 1000, respectively, matching those measured in the fluorescent reference strain.

Data extraction from colonies

Colonies are manually segmented in Fiji [1], with all of the subsequent steps carried out in Python. To estimate the mean colony radius, we calculate the distance of each point on the perimeter from the centroid using $\sqrt{(x - centroid_x)^2 + (y - centroid_y)^2}$. The centroid positions are estimated using OpenCV [4]. To extract fluorescence and brightfield values, we split the colonies in 360 sections, each 1° large. For each section, a radius is drawn and gray values are extracted along the radius for each of the four imaging channels (1 brightfield and 3 fluorescence channels).

Growth rate extraction

To extract growth rates from fluorescence time courses, after having observed that fluorescence reflects biomass well (SI Fig. 4), we compute the mean fluorescence values from illumination corrected boxes. Each of the resulting growth curves is independently fitted using [5] to extract the maximum growth rate. The ranges of the fitting parameters are: amplitude -5,5; flexibility -6,2; error -5,2.

Biomass to fluorescence relationship

To test whether fluorescence is a good proxy for biomass across various growth stages and nutritional conditions, we extract fluorescence values from mother machine pistons. Cells in stationary phase are loaded in the channels and allowed to grow to exponential phase. We examine 13 FOVs or more for *P. aeruginosa* and *S. aureus*, with around 70 pistons each and 42 for *C. albicans* with 35 pistons each. We did not perform single cell segmentation. Instead, fluorescence images are segmented using the adaptiveThreshold function with the mean thresholding method (block size = 71, constant = 4) of OpenCV [4]. We then apply global thresholding using Otsu's method. Segmented images are the result of the intersection of the two methods, to which 3 rounds of median blurs are applied. Fluorescence per time is calculated as the total mean of the fluorescence signal per pixel.

Single cell segmentation

The initial titers of cells in the geometrical alveoli mimics are extracted using a custom-made segmentation algorithm in Python. Using OpenCV [4], images are thresholded using the adaptiveThreshold function with the mean thresholding method. Contours are extracted from the resulting binary images and filtered for size to eliminate noise and debris. To separate large cell masses, the solidity of the contours is evaluated by estimating their hull size and split recursively through the center when a certain size limit is exceeded. This is sufficient to differentiate single *C. albicans* cells, but for *S. aureus* and *P. aeruginosa* we add a further splitting step that counts the number of cells within larger, still unsplit blobs. For *C. albicans*, before finding the final contours and quantifying their characteristics (position and sizes), we perform a dilation step to fill small gaps. The *P. aeruginosa* strain used produces the dimmest fluorescence signal per cell and thus its analysis requires some additional steps. Each image is copied twice, and the resulting three images (A, B and C) undergo slightly different processing steps: "A" is thresholded as before; "B" is smoothed using the

Gaussianblur method before thresholding, and "C" is contrast-enhanced using Contrast Limited Adaptive Histogram Equalization. The foregrounds of the three images are combined and further processed for contour individuation as done for *S. aureus*. We test the efficacy of this approach by benchmarking the numbers of cells segmented against counts obtained through visual inspection obtaining F1-scores equal or above 95% (SI Table 1).

Simulations

Simulations utilize an agent-based model based on [6], in which cells grow in a two-dimensional plane. Bacterial cells are designated as a single species to represent both PA and SA; symbolically, species $S = PA - SA$. *Candida* hyphae are represented by chains of linked cells, of species $S = CA$ (SI Fig. 8). Cells grow linearly from an initial length ℓ_S with an average growth rate μ_S, while cell diameters d_S are constant. The instantaneous length of each cell i is $\ell_i(\Delta t) = \ell_S + \mu_i \Delta t_i$ at a time Δt_i since division for an individual growth rate μ_i drawn uniformly from $(\mu_S/2,\ 3\mu_S/2)$ at division. Cells grow until they reach a set division length $\ell(\Delta t) = \ell^*_S$, at which point they divide into two daughters of equal length. Daughter cells inherit their mother's orientation with small perturbations.

The position r_i and orientation u_i evolve according to overdamped equations of motion $\dot{r}_i = \frac{1}{\ell_i \zeta_S} F_i \quad ; \quad \dot{u}_i = \frac{12}{\ell_i^3 \zeta_S} \tau_i$, where ζ_S is the friction per unit length, and F_i and τ_i are the net force and torque on the ith cell [6, 7]. Cells are simulated as spherocylinders that interact through steric pair potentials and are subject to drag $F_i^{drag} = -\zeta_S \ell_i \dot{r}_i$ and $\tau_i^{drag} = -\zeta_S \ell_i^3 \dot{u}/12$, which approximates drag as orientation-independent and proportional to cell length [8]. All other forces and torques result from pair potentials U_{ij} as $F_{ij} = -\nabla U_{ij}$. Steric potentials between spherocylinders i and j are modelled via a purely repulsive Lennard-Jones potential [9] $\mathrm{U}_{ij}^{steric} = \{ 4\,\epsilon \left[\left(\frac{d_{ij}}{r_{ij}}\right)^{12} - \left(\frac{d_{ij}}{r_{ij}}\right)^{6} \right] + \epsilon for r_{ij} < 2^{1/6} d_{ij}$
$0 otherwise$, with repulsion strength ϵ, characteristic size d_{ij} set to the mean diameter of the pair of cells and the separation r_{ij} calculated between two fictitious spheres located at the points of closest approach along the axes of symmetry of the two spherocylinders.The torque on cell i due to contact with j is $\tau_{ij}^{steric} = (p_{ij} - r_i) \times F_{ji}^{steric}$, where the location of the force p_{ij} is taken to be at the surface of cell i closest to the axis of cell j.

In addition, each *C. albicans* cell has bonds that connect it to the extreme ends of the axes of adjacent cells in the hyphal chain. The bond length l_{ij} obeys a potential $U_{ij}^{bond} = \frac{K}{2}\left(l_{ij}^{bond} - d^{bond}\right)^2$ for a compression modulus K and rest length d^{bond}, which is set to be slightly smaller than d_S for *Candida* to avoid the smaller bacterial cells penetrating the chain.

The bending energy is $U_{ij}^{bend} = \frac{B}{2}\theta_{ij}^2$ for bending stiffness B, with θ_{ij} the angle between the axes of neighboring segments on the chain. The torques result from applying the forces at the ends of the cell axes. Hyphae filaments divide when the number of segments reaches a maximum value of $N = 50$; at which point, the middle bond is removed.

The bacterial parameters ($S = PA - SA$) are $d_{PA-SA} = 1.17$ μm, $\ell_{PA-SA} = 3$ μm, $\ell^*_{PA-SA} = 6$ μm, $\mu_{PA-SA} = 5$ μm/h, $\zeta_{PA-SA} = 200$ Pa·h. The parameters for *C. albicans* ($S = CA$) are $d_{CA} = 2.36$ μm, $\ell_{CA} = 4$ μm, $\ell^*_{CA} = 6$ μm, $\mu_{CA} = 5$ μm/h, $\zeta_{CA} = 200$ Pa·h. The hyphae compression modulus is $K = 4 \times 10^{-6}$ N/m, bending stiffness $B = 4 \times 10^{-17}$ N·m and the rest length $d^{bond} = 0.65\ d_{CA}$. For both bacteria and *C. albicans*, the steric repulsion is set to $\epsilon = 5.5 \times 10^{-12}$ N·m. A single microfluidic alveoli mimic is simulated as a 2D square microchamber of length $L = 150\mu$m. The microchamber is constructed of three planar walls and a planar opening. The walls are modeled as repulsive potentials that increase quadratically with the degree of cell/segment overlap with modulus $K^{wall} = 4 \times 10^6$ N/m. When both ends of a cell pass the opening plane, it is instantaneously removed from the simulation. This simulates the loss of cells to flow in the main microfluidic channel.

The numerical experiments make a number of simplifying assumptions. For example, the friction is modeled as isotropic and linearly dependent on length, while any cell-cell and cell-substrate interactions beside friction are neglected. Likewise, the bending rigidity is taken to be linear in the number of segments. Simulations must also make assumptions about the initialization, timescales of interest and dimensionality. Cells are either placed in the center of the mimics (Fig. 5) or homogeneously and isotopically throughout (SI Fig. 7), rather than being deposited by advective flow. The simulations run for $\sim$ 10h and the geometry in simulations is strictly two dimensional, as opposed to the experiments (Fig. 2B). While these various simplifications in the agent-based model may lead to minor quantitative discrepancies, their simplicity allows us to directly test the hypothesis that eccentric hyphae growth allows *C. albicans* to reach the closed ends of the confinements and establish temporary spatial structure.

Supplementary Figures

Species	Automatic/manual count	F1-score
P. aeruginosa	1145/1042, 1017/1019, 966/1179	0.95
S. aureus	1135/1140, 986/917, 743/796	0.96
C. albicans	17/17, 4/4, 17/16, 12/11	0.98

Table 1: **Benchmarking of segmentation algorithms.** The manual count is taken as the true count.

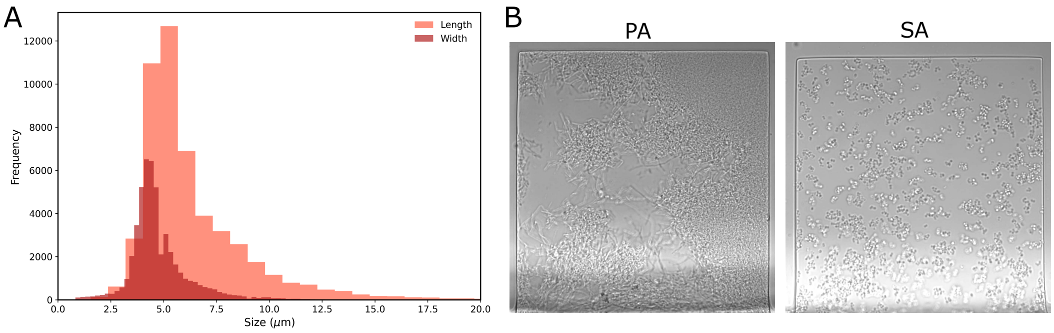

Figure 1: **Analysis of the layered occupancy of the chambers.** a) Size distribution of *C. albicans* at the seeding. Measures where extracted via segmentation of the first time point from 9 PA-SA-CA experiments (900 microchambers). b) representative brightfield images showing multi-layered growth of *P. aeruginosa* (left) and *S. aureus* (right).

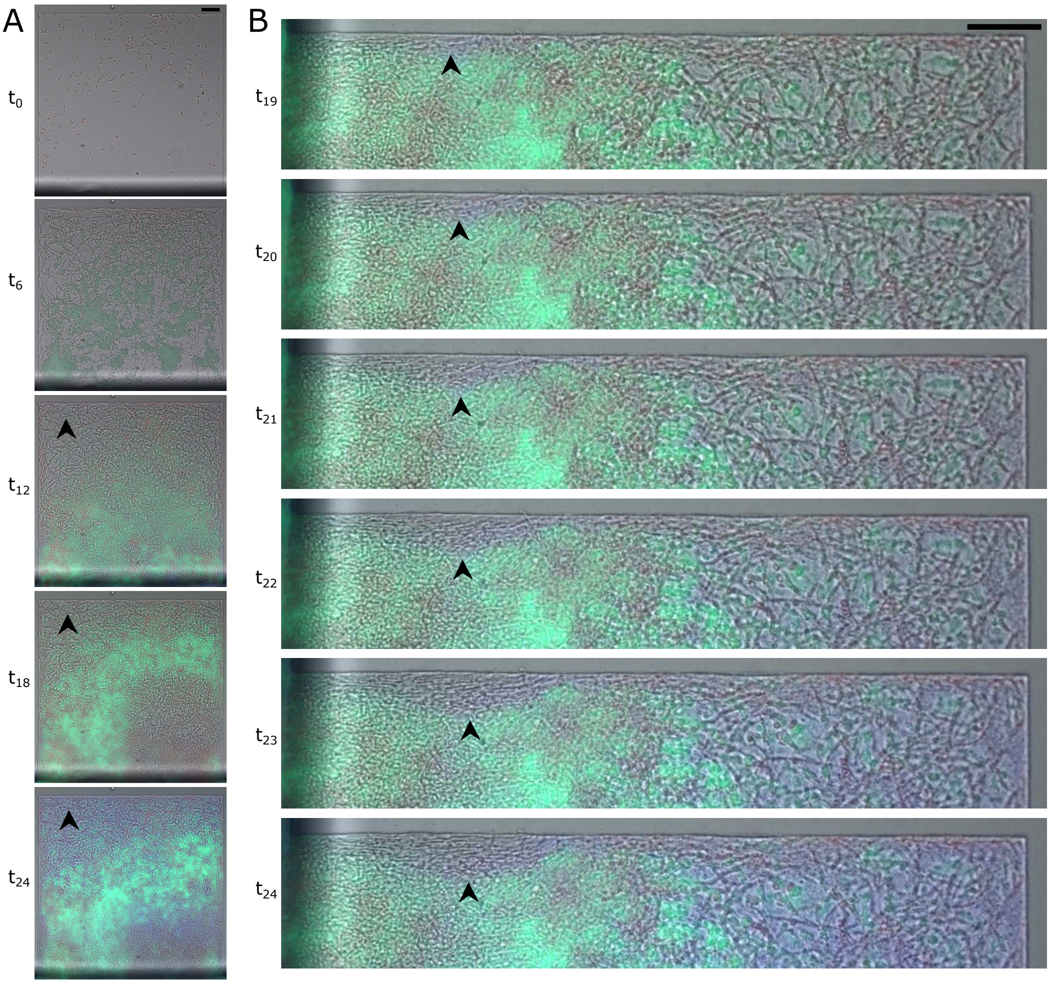

Figure 2: **Analysis of the community's growth dynamics in boxes with increased volume but unchanged height.** a) Time course of the PA-SA-CA polymicrobial community in a box with side length 500 μm and height 8 μm. The *C. albicans* at the closed end of the microenvironment stops growing. The arrowheads point to an area where growth has clearly stopped. b) zoom-in from (a) tilted 90° to the right showing that in the proximity of the opening, *C. albicans* conquers the edge and excludes bacteria. Black arrows indicate the area of progressive growth. Scale bars =50 μm.

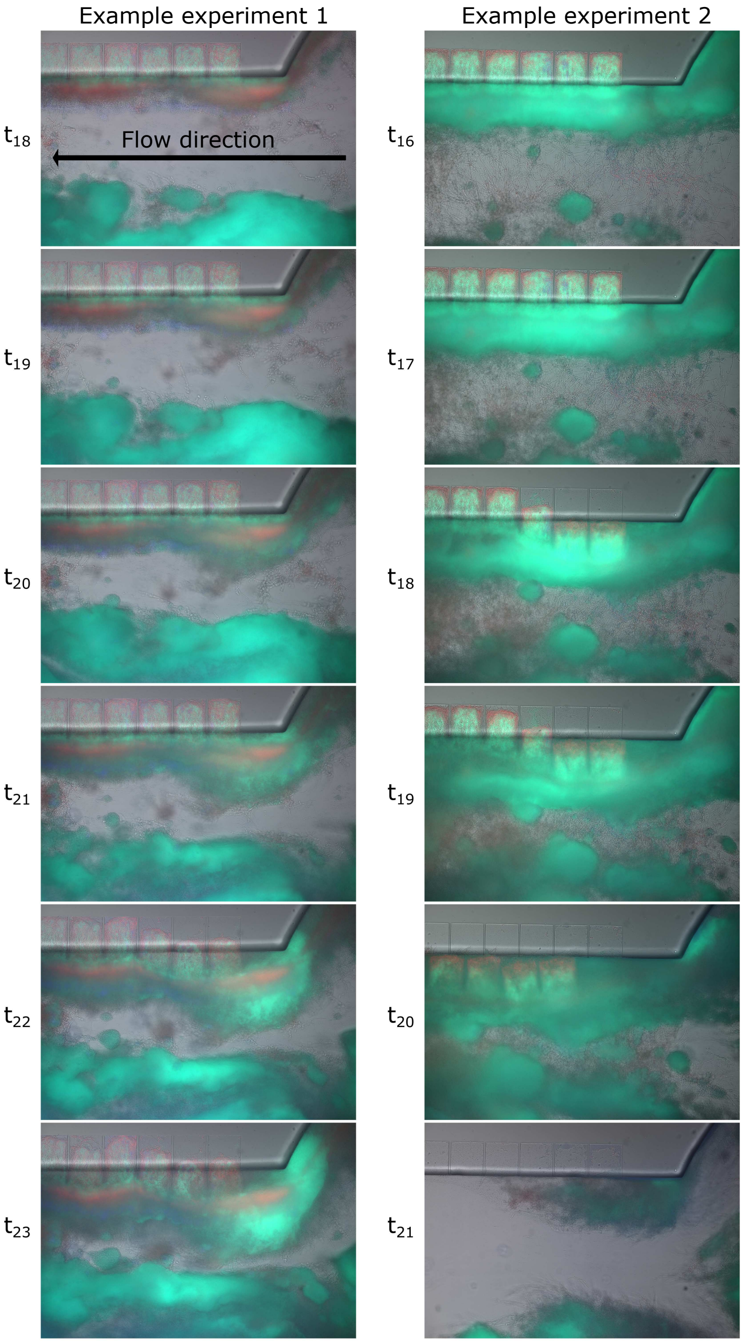

Figure 3: **Biofilm behavior in the main channel.** After 18 to 20 hours, growth in the main channel becomes substantial and the resulting biomass, further to potentially decreasing perfusion of nutrients in the boxes, can be peeled off by the flow taking away box contents. For this reason we limit our observations to the first 20 hours. Two examples from independent experiments are given (left and right). The size of the boxes' sides is 150 μm and provides scale.

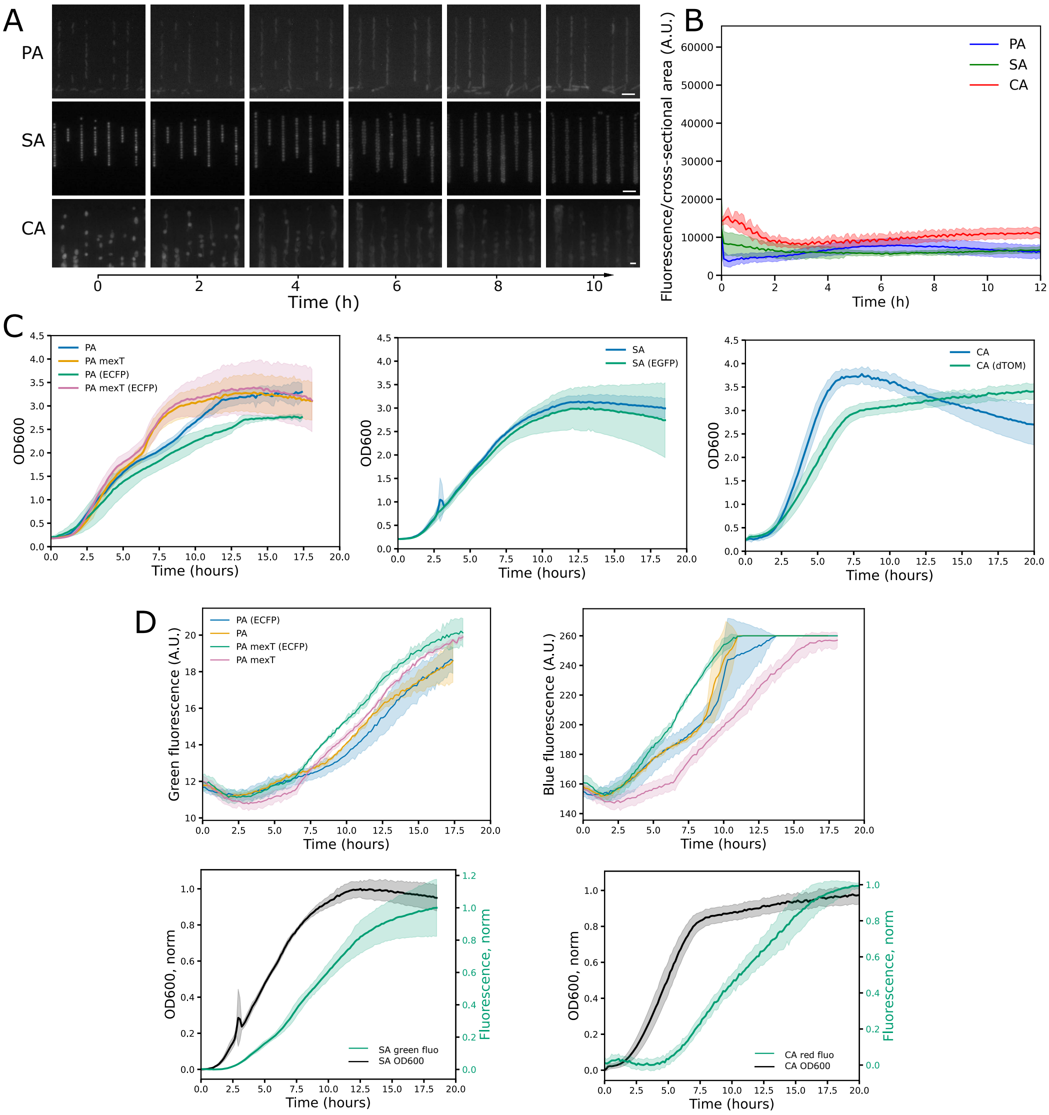

Figure 4: **Evaluation of fluorescence as a proxy for biomass in our strains.** a) Fluorescence and biomass (z-limited cross-sectional area) were extracted from timelapse experiments in mother machines in ASM. b) Average fluorescence per pixel from cells from 13 fovs or more for *P. aeruginosa* and *S. aureus*, with around 70 pistons each and 42 for *C. albicans* with 35 pistons each plotted in the camera sensitivity range. The shaded areas indicate the standard deviation. *P. aeruginosa* = blue, *S. aureus* = green, *C. albicans* = red. Scale bar = 5μm. c) Expression of fluorescent proteins causes small fitness reductions in the members of the community. Optical density readings obtained in a plate reader. Curves are averages from at least 3 replicates, the shaded areas show the standard deviation. d) Bulk assays are unsuitable for the characterization of fluorescence per biomass. Top: in bulk, pigment production by all of the tested *P. aeruginosa* strains masks ECFP fluorescence. Bottom: different sensitivity ranges, scattering, and inner filter phenomena make difficult the comparison between biomass and fluorescence in bulk assays outside the period of exponential growth.

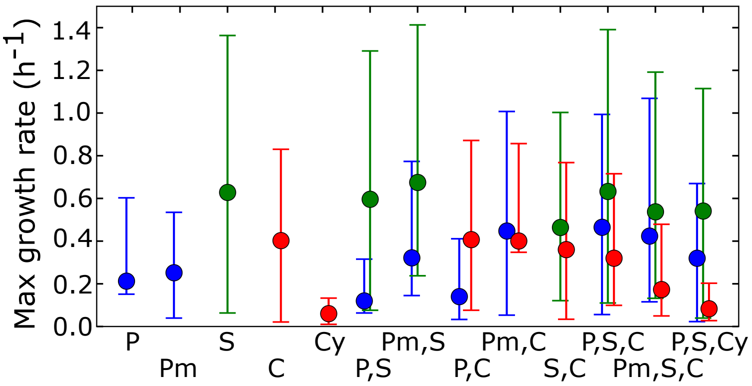

Figure 5: **Comparisons between growth rates extracted from geometrical alveoli mimics.** P, S, and C are short for PA, SA, and CA. *Pm* indicates *P. aeruginosa mexT* mutant, *Cy* indicates *C. albicans hgc1*Δ/Δ mutant. *P. aeruginosa* = blue, *S. aureus* = green, *C. albicans* = red. Each average is extracted from at least 300 microenvironments from at least 3 independent experiments, the error bar shows the standard deviation.

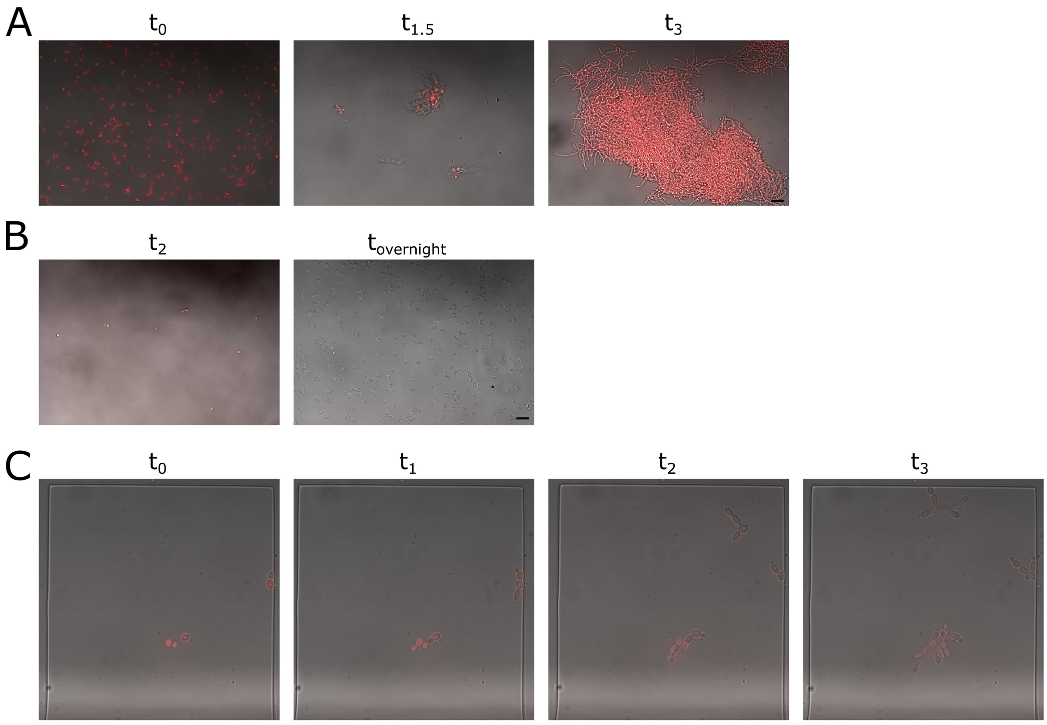

Figure 6: **Assay of whether hyphal transition is due to contact with the surface.** a) Time course of *C. albicans* cells grown in shake flasks in ASM at 37°C. In ASM, surfaces are not necessary for hyphae formation. Scale bar = 20 μm. b) *C. albicans* growth in YPD. Scale bar = 20 μm. c) *C. albicans* growth in YPD in microenvironments and production of pseudohyphae: the microenvironment's surface is not sufficient to induce hyphae formation. The size of the boxes' sides is 150 μm and provides scale.

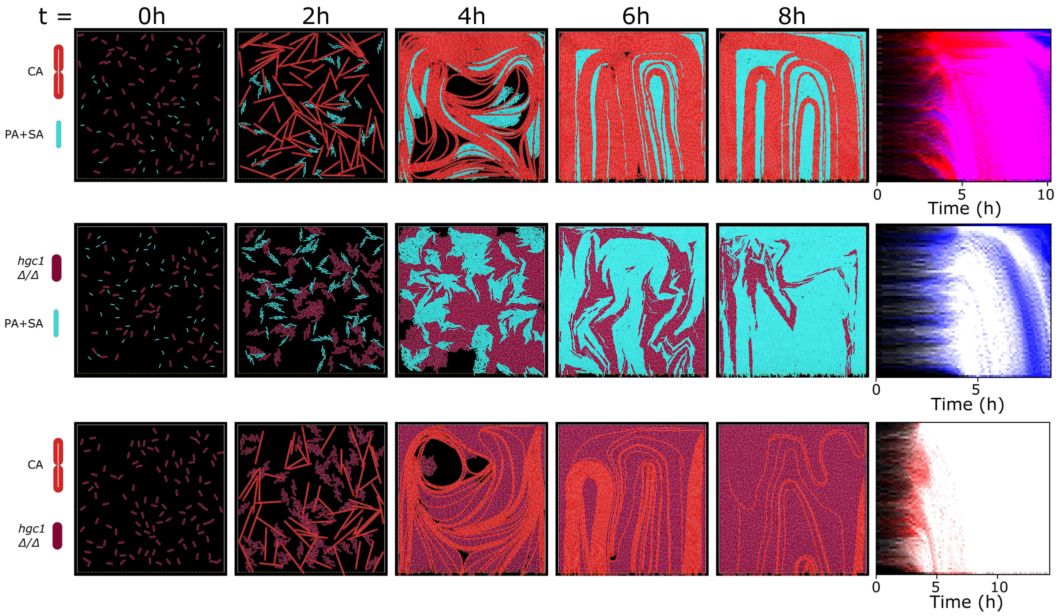

Figure 7: **2D simulations to assay the impact of starting position and number of microbes.** Top: hyphae-competent *C. albicans* initially positions itself at the edges, dislodging bacteria and hence exhibiting spatial structure (6h). However, mechanical forces then push bacteria behind the last layer of *C. albicans*, which typically founds a population that eventually takes over the niche, as shown by the kymograph. Centre: the yeast-locked mutant *hgc1*Δ:Δ does not give rise to spatial organization. In the kymograph, the mutant is given in white. Bottom: in 2D, a yeast-locked strain can displace a hyphae-competent one, eventually dominating the space. This shows that the spatial structure observed in Fig. 5H disappears if the cells are initialized homogeneously throughout the geometrical alveoli mimic. In the kymograph, the mutant is given in white. Kymographs are obtained from 10 simulations, each of 112 cells initialized homogeneously and isotropically throughout the niche with ratios of 3:1 for the top row and 1:1 for the middle and bottom rows

.

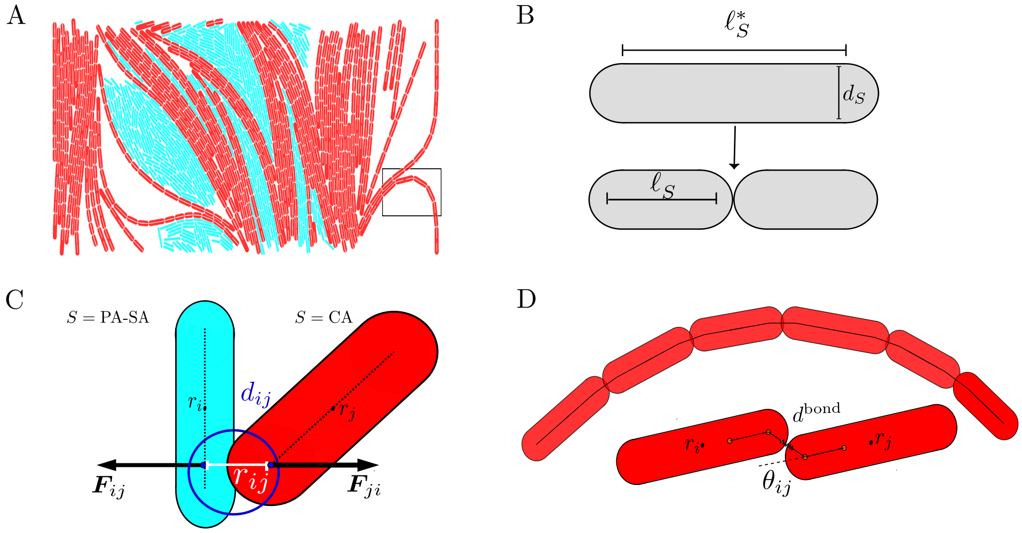

Figure 8: **Schematic description of the species used in the simulations.** a) Snapshot illustrating bacteria (S=PA-SA) in cyan and fungal hyphae (S=CA) in red. b) Schematic for division events of both bacteria cells and hyphae segments. Cells/segments increase their length ℓ linearly in time until they reach a critical length, ℓ_S^*, at which point they divide into two daughters of length ℓ_S and diameter d_S. c) Steric repulsion forces F_{ij} and F_{ji} between cells/segments i and j results from the nearest distance r_{ij}. The characteristic diameter d_{ij} is the average diameter of the interacting segments. D) Magnified view of hyphal segments within the highlighted area (black rectangle in A), showing segments connected by bonds (top). A schematic (bottom) demonstrates two segments chained together. Each CA segment in a chain is connected via bonds linking the extreme ends of adjacent cells in the hyphal chain. The bonds have a rest length d^{bond} and θ_{ij} is the angle between the axes of neighbouring segments.

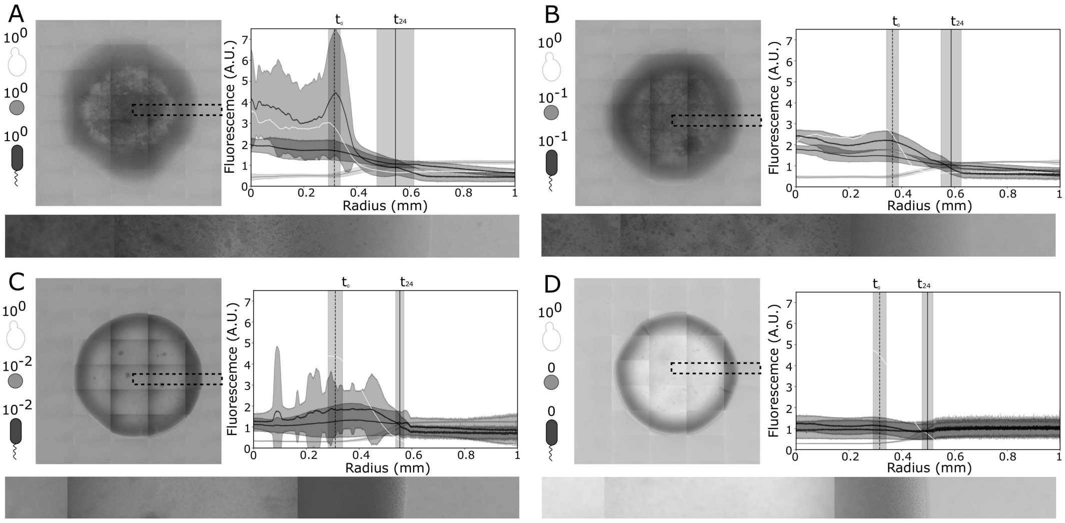

Figure 9: **Red colour channel of the Main Fig. 1 given in grayscale.**

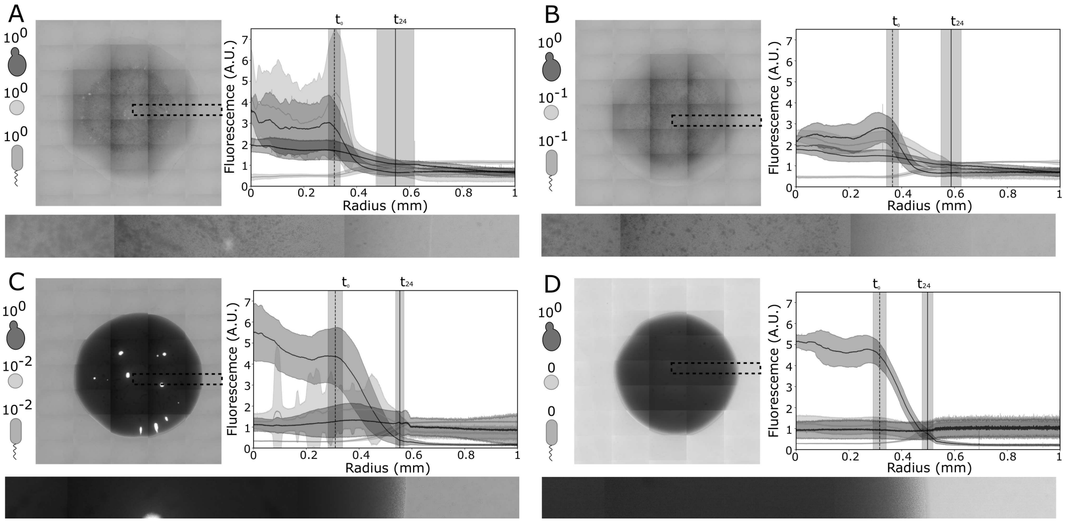

Figure 10: **Green colour channel of the Main Fig. 1 given in grayscale.**

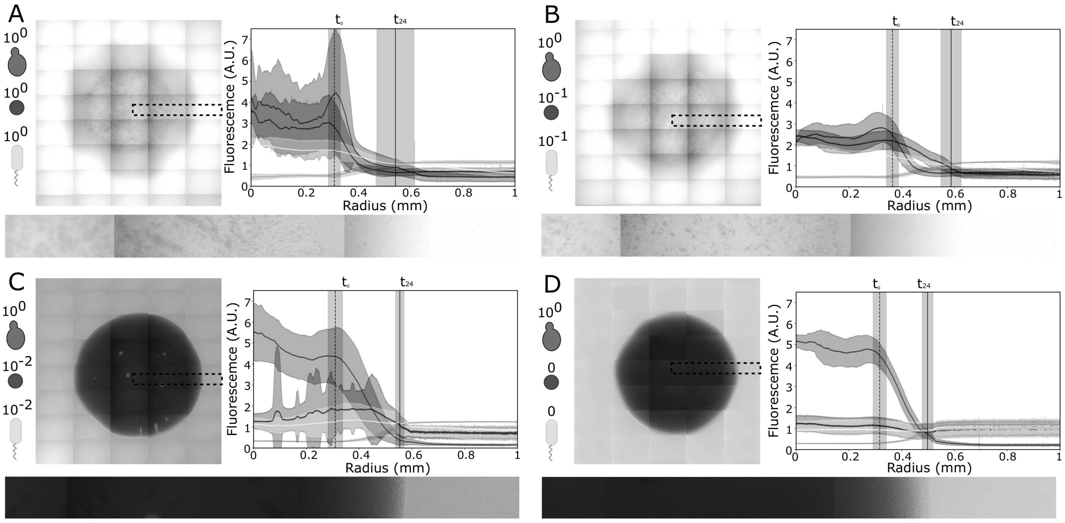

Figure 11: **Blue colour channel of the Main Fig. 1 given in grayscale.**

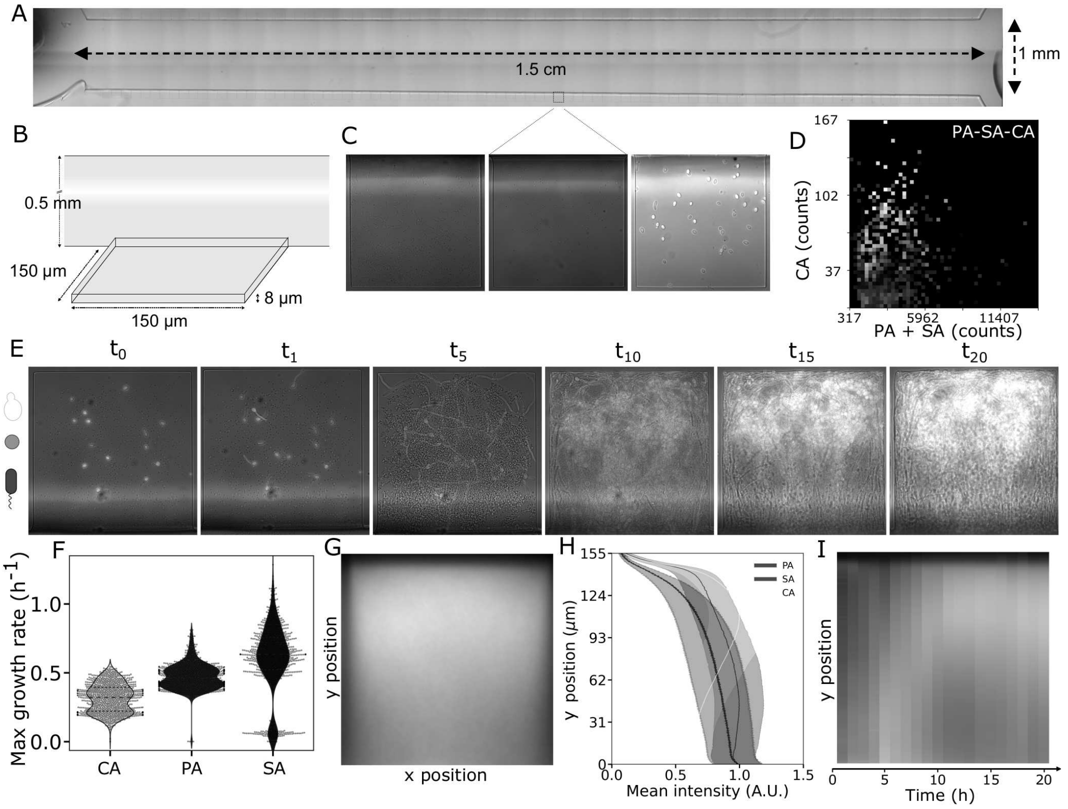

Figure 12: **Red colour channel of the Main Fig. 2 given in grayscale.**

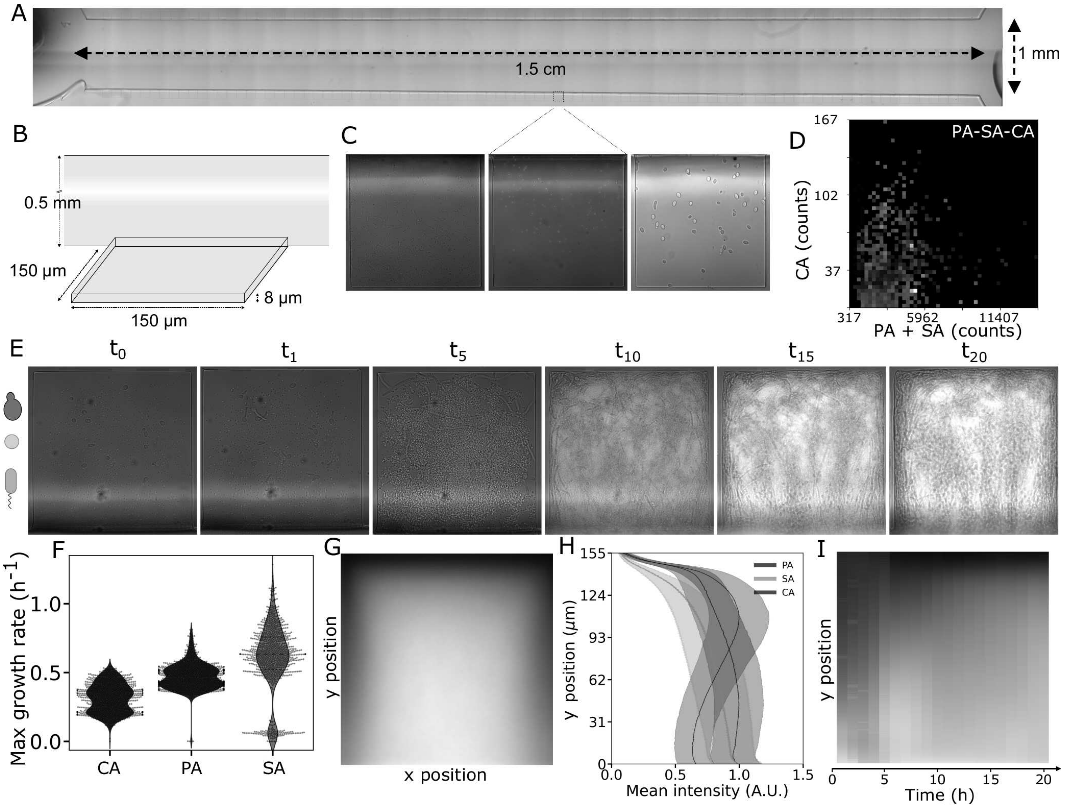

Figure 13: **Green colour channel of the Main Fig. 2 given in grayscale.**

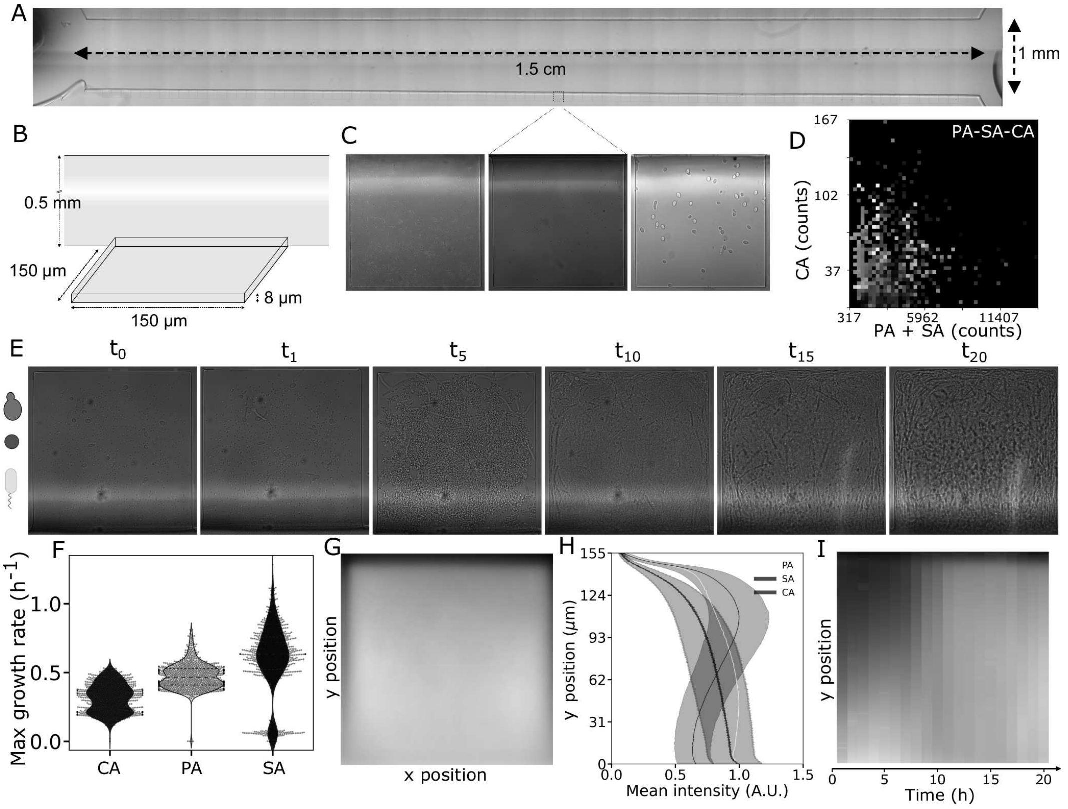

Figure 14: **Blue colour channel of the Main Fig. 2 given in grayscale.**

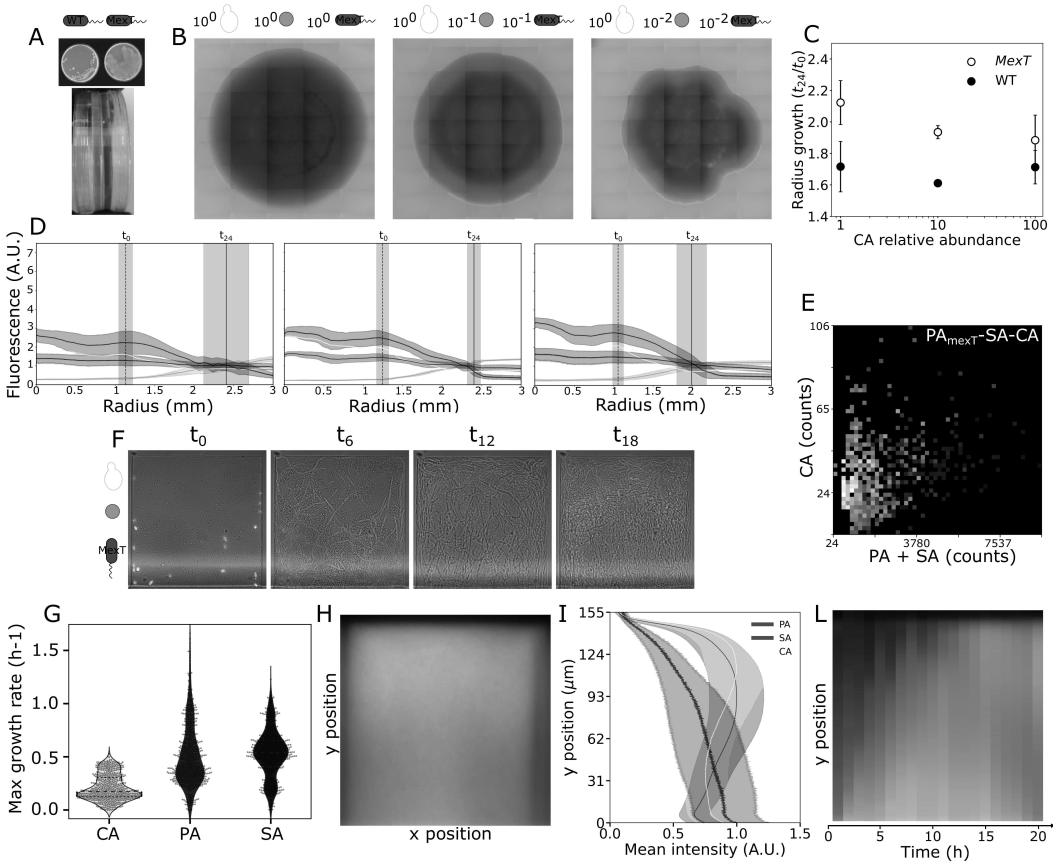

Figure 15: **Red colour channel of the Main Fig. 3 given in grayscale.**

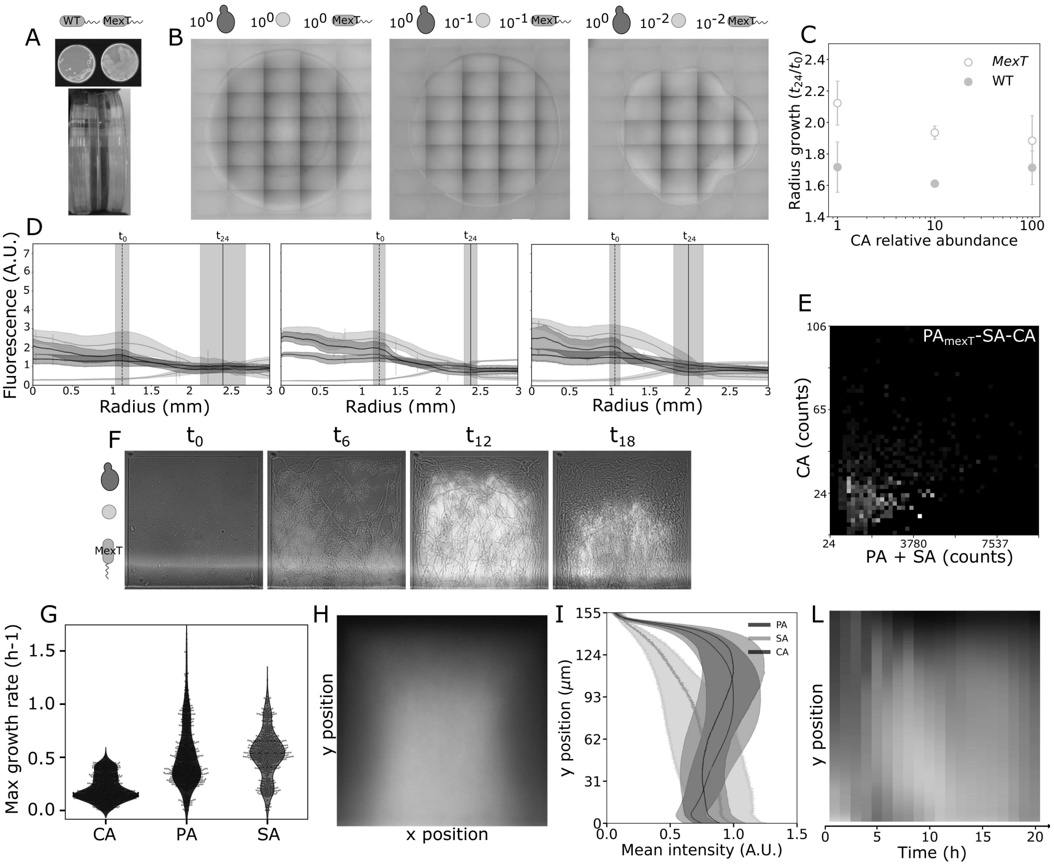

Figure 16: **Green colour channel of the Main Fig. 3 given in grayscale.**

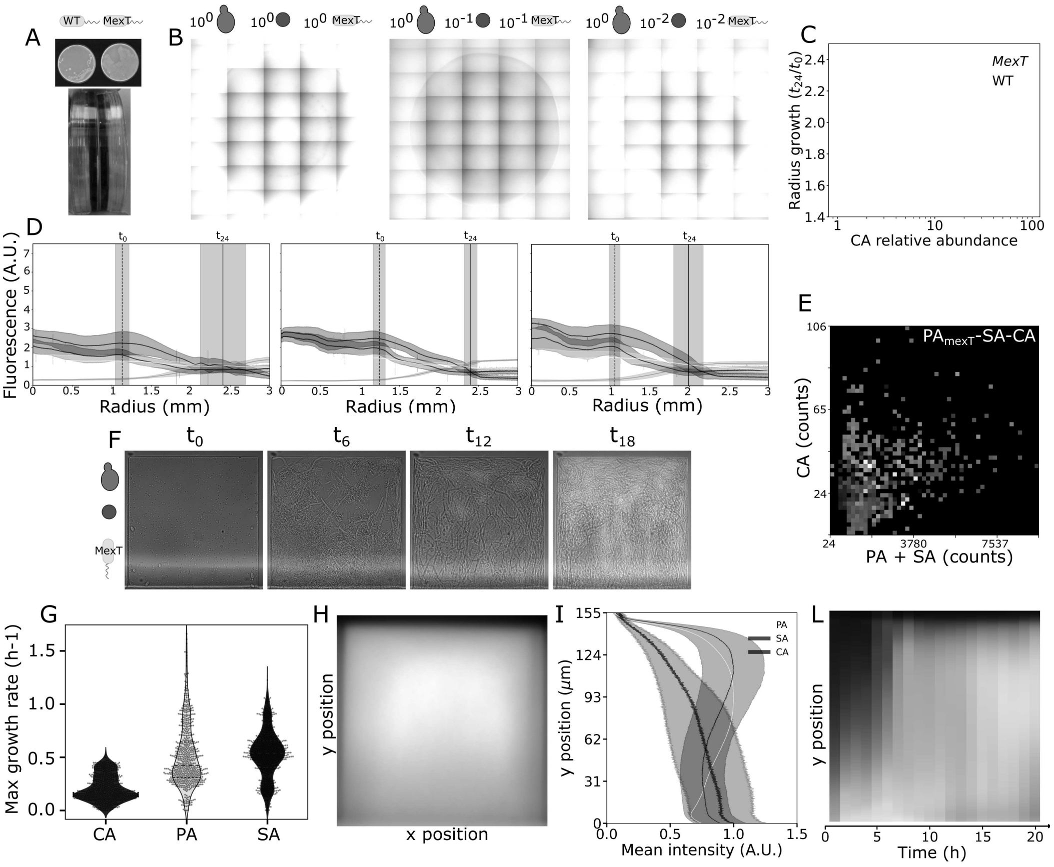

Figure 17: **Blue colour channel of the Main Fig. 3 given in grayscale.**

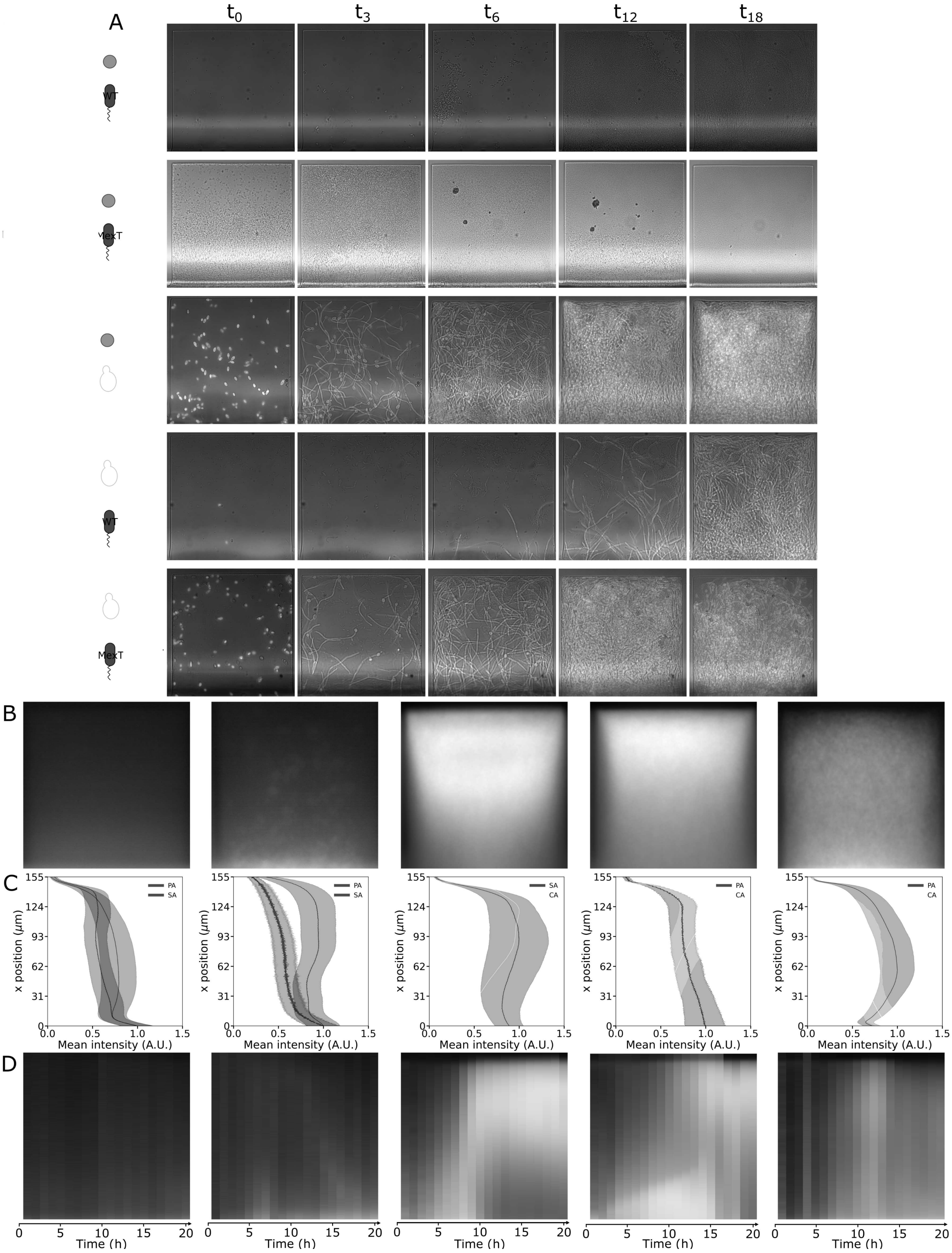

Figure 18: **Red colour channel of the Main Fig. 4 given in grayscale.**

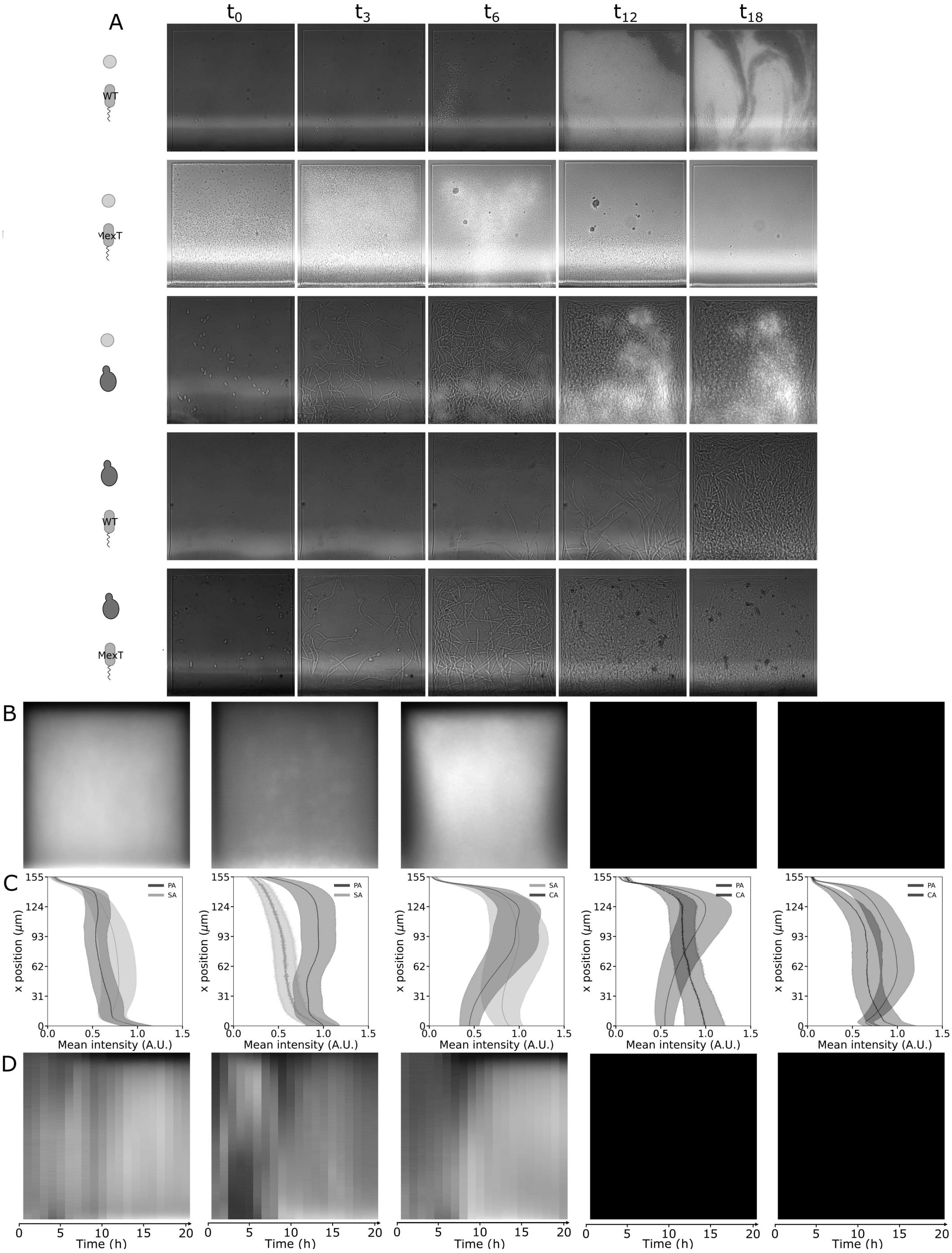

Figure 19: **Green colour channel of the Main Fig. 4 given in grayscale.**

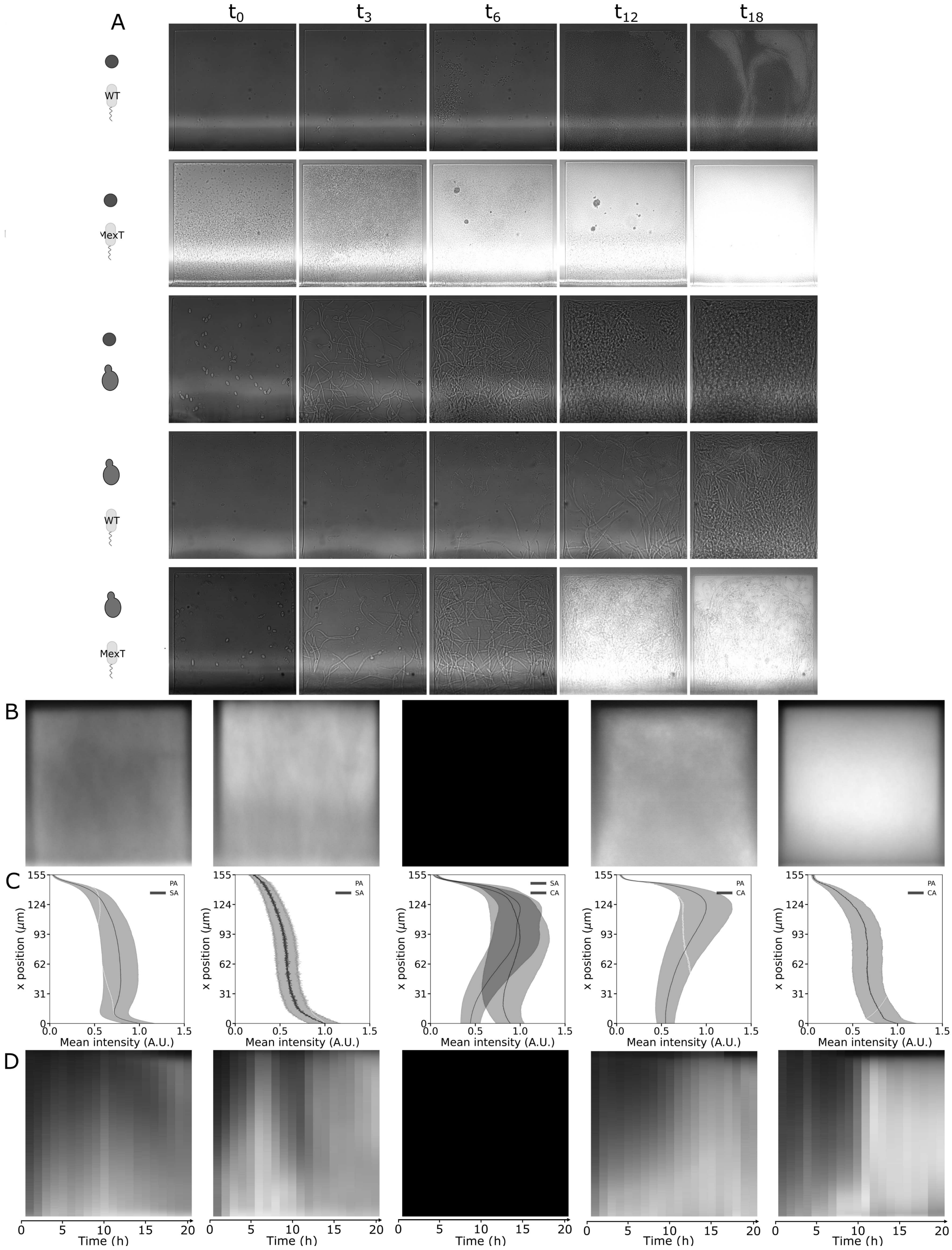

Figure 20: **Blue colour channel of the Main Fig. 4 given in grayscale.**

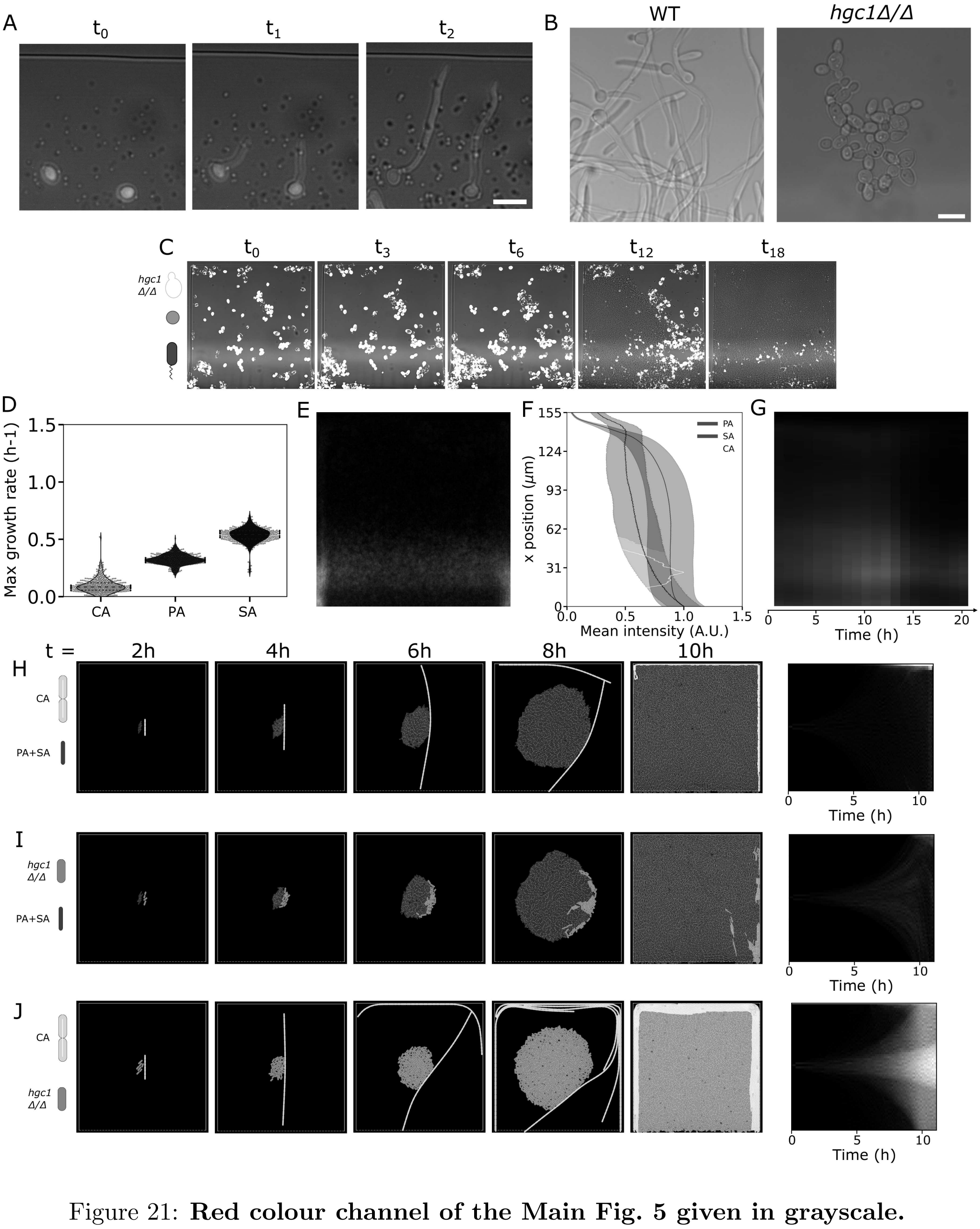

Figure 21: **Red colour channel of the Main Fig. 5 given in grayscale.**

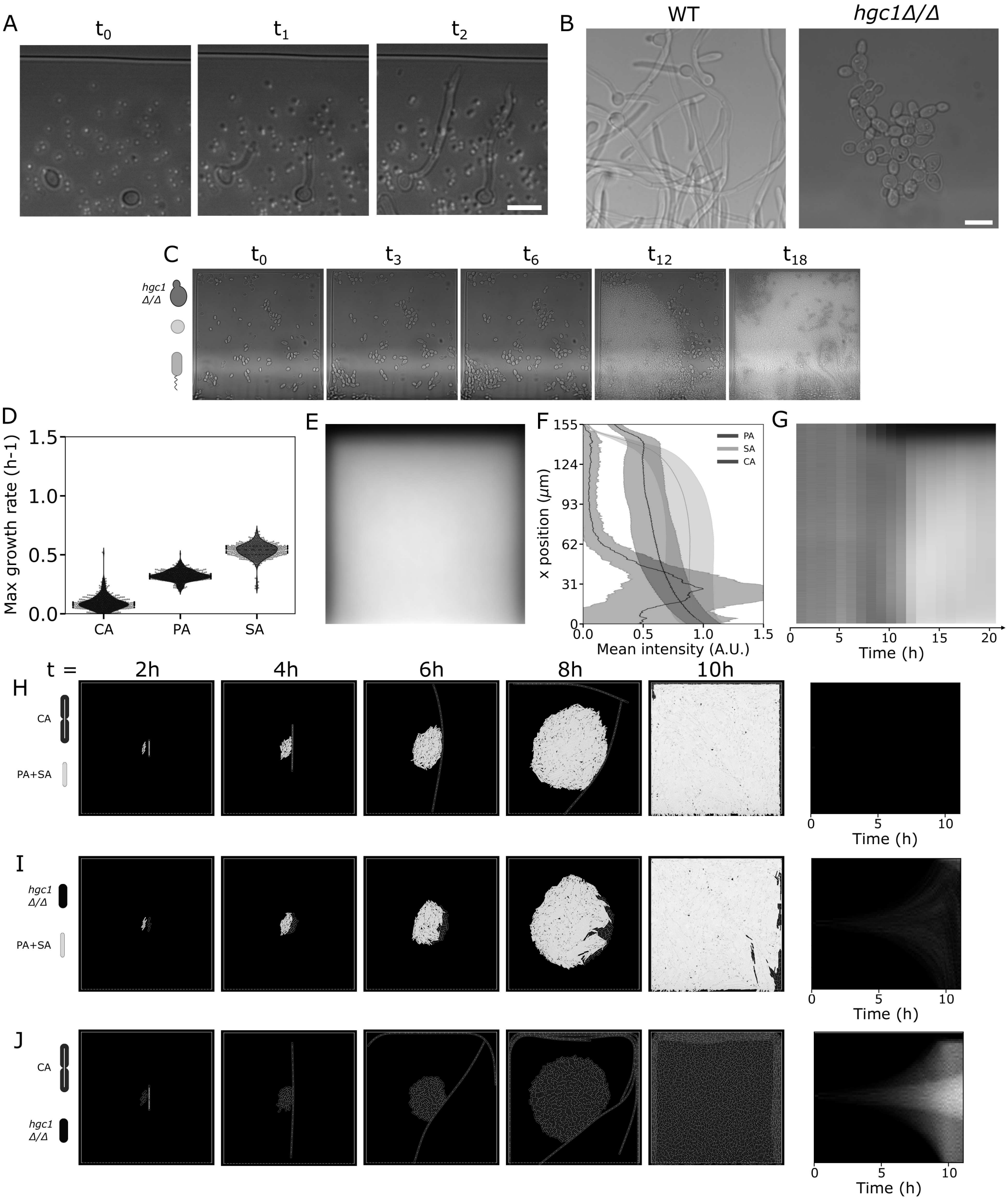

Figure 22: **Green colour channel of the Main Fig. 5 given in grayscale.**

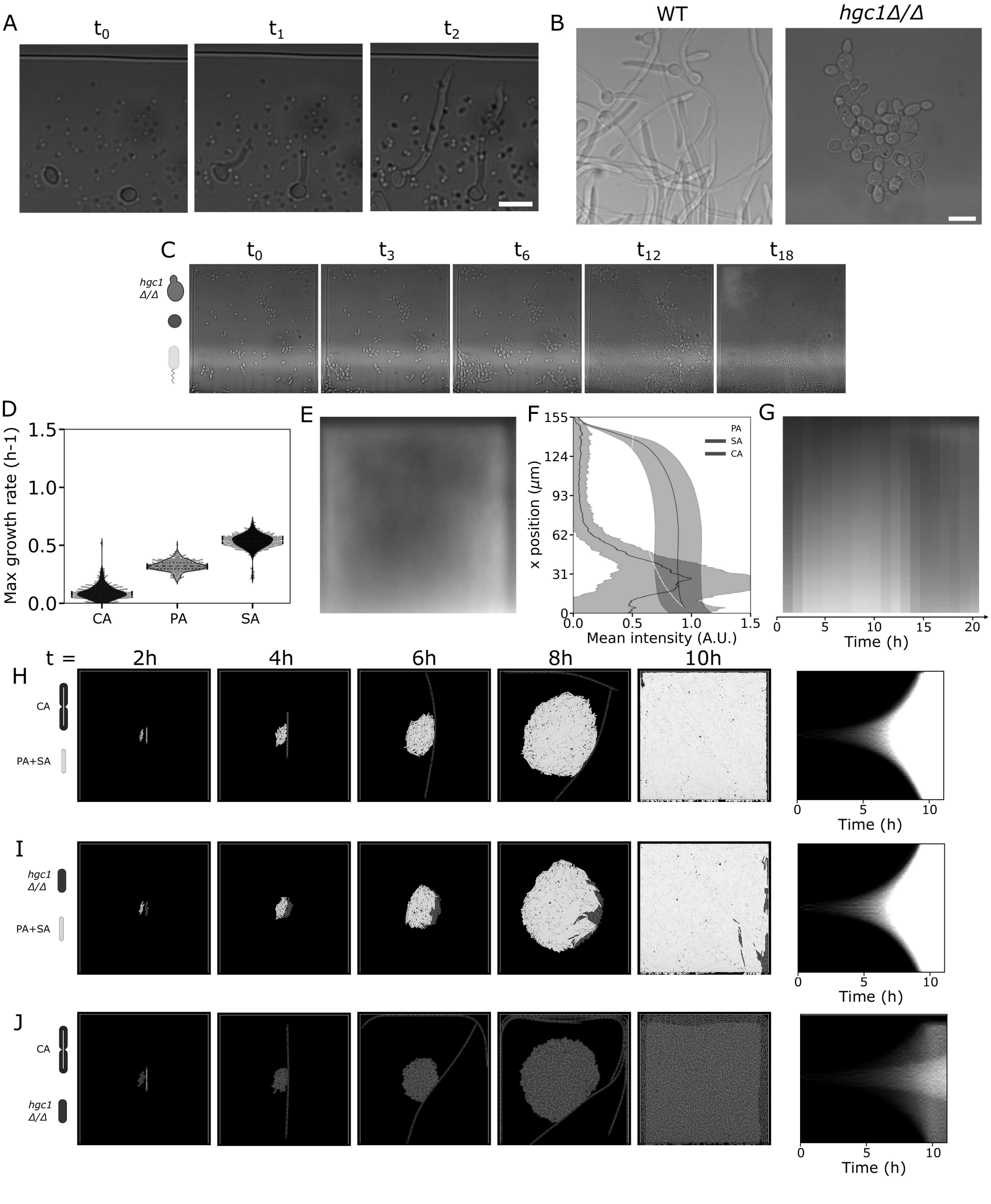

Figure 23: **Blue colour channel of the Main Fig. 5 given in grayscale.**

SI Video 1

Example community of *P. aeruginosa* (blue), *C. albicans* (red), and *S. aureus* (green) grown in geometrical alveoli mimics. These images are part of the dataset pertaining to main Fig. 2.

SI Video 2

Example community of *P. aeruginosa* (blue) carrying a frameshift mutation in *mexT*, *C. albicans* (red), and *S. aureus* (green) grown in geometrical alveoli mimics. These images are part of the dataset relevant to main Fig. 3.

SI Video 3

Example community of *P. aeruginosa* (blue) and *S. aureus* (green) grown in geometrical alveoli mimics. These images are part of the dataset pertaining to main Fig. 4.

SI Video 4

Example community of *P. aeruginosa* (blue) carrying a frameshift mutation in *mexT* and *S. aureus* (green) grown in geometrical alveoli mimics. These images are part of the dataset pertaining to main Fig. 4.

SI Video 5

Example community of *S. aureus* (green) and *C. albicans* (red) grown in geometrical alveoli mimics. These images are part of the dataset pertaining to main Fig. 4.

SI Video 6

Example community of *P. aeruginosa* (blue) and *C. albicans* (red) grown in geometrical alveoli mimics. These images are part of the dataset pertaining to main Fig. 4.

SI Video 7

Example community of *P. aeruginosa* (blue) carrying a frameshift mutation in *mexT* and *C. albicans* (red) grown in geometrical alveoli mimics. These images are part of the dataset pertaining to main Fig. 4.

SI Video 8

Example community of *P. aeruginosa*, *C. albicans hgc1*Δ*:*Δ (false-coloured red), and *S. aureus* grown in geometrical alveoli mimics. These images are part of the dataset pertaining to main Fig. 5.

SI Video 9

Example simulation of a community of *C. albicans* (red) and "bacteria" (cyan). These images are part of the dataset pertaining to main Fig. 5.

SI Video 10

Example simulation of a community of *C. albicans hgc1*Δ*:*Δ (deep red) and "bacteria" (cyan). These images are part of the dataset pertaining to main Fig. 5.

SI Video 11

Example simulation of a community of *C. albicans* (red) and *C. albicans hgc1*Δ*:*Δ (deep red). These images are part of the dataset pertaining to main Fig. 5.

References

[1] J. Schindelin, I. Arganda-Carreras, E. Frise, et al. "Fiji: an open-source platform for biological-image analysis". *Nature Methods* 2012;9:676–682. 10.1038/nmeth.2019.

[2] S. Berg, D. Kutra, T. Kroeger, et al. "ilastik: interactive machine learning for (bio)image analysis". *Nature Methods* 2019;16:1226–1232. 10.1038/s41592-019-0582-9.

[3] S. Preibisch, S. Saalfeld, and P. Tomancak "Globally optimal stitching of tiled 3D microscopic image acquisitions". *Bioinformatics* 2009;25:1463–1465. 10.1093/bioinformatics/btp184.

[4] G. Bradski "The OpenCV Library". *Dr. Dobb's Journal of Software Tools* 2000.

[5] P. S. Swain, K. Stevenson, A. Leary, et al. "Inferring time derivatives including cell growth rates using Gaussian processes". *Nature Communications* 2016;7:13766. 10.1038/ncomms13766.

[6] Z. You, D. J. G. Pearce, A. Sengupta, et al. "Geometry and Mechanics of Microdomains in Growing Bacterial Colonies". *Physical Reveview X* 2018;8:031065. 10.1103/PhysRevX.8.031065.

[7] X. Jin and J. S. Marshall "Influence of Cell Interaction Forces on Growth of Bacterial Biofilms". *Physics of Fluids* 2020;32:091902. 10.1063/5.0021126.

[8] F. D. C. Farrell, O. Hallatschek, D. Marenduzzo, et al. "Mechanically Driven Growth of Quasi-Two-Dimensional Microbial Colonies". *Physical Review Letters* 2013;111:168101. 10.1103/PhysRevLett.111.168101.

[9] G. W. Slater, C. Holm, M. V. Chubynsky, et al. "Modeling the separation of macromolecules: A review of current computer simulation methods". *Electrophoresis* 2009;30:792–818. 10.1002/elps.200800673.
